# The Effect of Boron on the Microstructure and Properties of Refractory Metal Intermetallic Composites (RM(Nb)ICs) Based on Nb-24Ti-xSi (x = 16, 17 or 18 at.%) with Additions of Al, Cr or Mo

**DOI:** 10.3390/ma14206101

**Published:** 2021-10-15

**Authors:** Tophan Thandorn, Panos Tsakiropoulos

**Affiliations:** 1 Department of Materials Science and Engineering, School of Science, Mae Fah Luang University, Chiang Rai 57100, Thailand; tophan@mfu.ac.th; 2 Department of Materials Science and Engineering, Sir Robert Hadfield Building, The University of Sheffield, Sheffield S1 3JD, UK

**Keywords:** Nb silicide-based alloys, high entropy alloys, complex concentrated alloys, refractory metal intermetallic composites, alloy design, intermetallics, solid solution, oxidation

## Abstract

This paper is about metallic ultra-high temperature materials, in particular, refractory metal intermetallic composites based on Nb, i.e., RM(Nb)ICs, with the addition of boron, which are compared with refractory metal high entropy alloys (RHEAs) or refractory metal complex concentrated alloys (RCCAs). We studied the effect of B addition on the density, macrosegregation, microstructure, hardness and oxidation of four RM(Nb)IC alloys, namely the alloys TT2, TT3, TT4 and TT8 with nominal compositions (at.%) Nb-24Ti-16Si-5Cr-7B, Nb-24Ti-16Si-5Al-7B, Nb-24Ti-18Si-5Al-5Cr-8B and Nb-24Ti-17Si-3.5Al-5Cr-6B-2Mo, respectively. The alloys made it possible to compare the effect of B addition on density, hardness or oxidation with that of Ge or Sn addition. The alloys were made using arc melting and their microstructures were characterised in the as cast and heat-treated conditions. The B macrosegregation was highest in TT8. The macrosegregation of Si or Ti increased with the addition of B and was lowest in TT8. The alloy TT8 had the lowest density of 6.41 g/cm^3^ and the highest specific strength at room temperature, which was also higher than that of RCCAs and RHEAs. The Nb_ss_ and T2 silicide were stable in the alloys TT2 and TT3, whereas in TT4 and TT8 the stable phases were the Nb_ss_ and the T2 and D8_8_ silicides. Compared with the Ge or Sn addition in the same reference alloy, the B and Ge addition was the least and most effective at 800 °C (i.e., in the pest regime), when no other RM was present in the alloy. Like Ge or Sn, the B addition in TT2, TT3 and TT4 did not suppress scale spallation at 1200 °C. Only the alloy TT8 did not pest and its scales did not spall off at 800 and 1200 °C. The macrosegregation of Si and Ti, the chemical composition of Nb_ss_ and T2, the microhardness of Nb_ss_ and the hardness of alloys, and the oxidation of the alloys at 800 and 1200 °C were also viewed from the perspective of the alloy design methodology NICE and relationships with the alloy or phase parameters VEC, δ and Δχ. The trends of these parameters and the location of alloys and phases in parameter maps were found to be in agreement with NICE.

## 1. Introduction

Research is underway worldwide to develop new metallic ultra-high temperature materials (UHTMs) to replace Ni superalloys in the hottest parts of future aero-engines to enable the latter to meet environmental and performance targets. Candidate metallic UHTMs include refractory metal (RM) intermetallic composites (RMICs), RM high entropy alloys (RHEAs) and RM complex concentrated alloys (RCCAs) (unnecessarily, in our opinion, another term has been added for RHEAs and RCCAs to further muddle the names used for metallic UHTMs, namely refractory chemically complex alloy (RCCAs)). The new materials must meet specific property targets (goals) for toughness, creep and oxidation resistance [1]. RMICs include materials based on Nb (RM(Nb)ICs, also known as Nb silicide-based alloys or Nb in situ composites) and Mo (RM(Mo)ICs). Recently, RM(Nb)ICs, RHEAs and RCCAs were compared in [1]. Some RM(Nb)ICs with B addition are also RCCAs [2] (i.e., RM(Nb)ICs/RCCAs). This paper is about RM(Nb)ICs with B addition and Ti/Si > 1.3 [1].

According to the alloy design methodology NICE (Niobium Intermetallic Composite Elaboration) [3], the alloying behaviour of RM(Nb)ICs and their phases, namely bcc Nb solid solution(s), M_5_Si_3_ silicides (M = RM = Mo, Ta or W, or M=TM (transition metal) = Cr, Hf or Ti), C14–NbCr_2_ based Laves and A15-Nb_3_X (X = Al, Ge, Si or Sn) compounds can be described using the parameters ΔH_mix_, ΔS_mix_, δ, VEC, Δχ, Ω (=T_m_ ΔS_mix_/|ΔH_mix_|) that are also used to describe the alloying of HEAs (see Appendix A). RM(Nb)ICs and their phases, and HEAs can be presented in maps of the above parameters [4,5,6,7,8,9]. Solid solution(s) RHEAs and RCCAs, and multiphase RCCAs (bcc solid solution(s) + M_5_Si_3_ silicide with/without Laves phase) can be presented in maps of aforementioned parameters together with RM(Nb)ICs and their bcc solid solution(s) [1]. The RM(Nb)ICs with B addition occupy a separate distinct area in the maps [1,2,5].

Boron in RM(Nb)ICs partitions to the bcc solid solution, the 5-3 silicide [4,6,10,11] and Nb_3_Si [11,12]. According to the Nb–Si–B phase equilibria, the 5-3 silicides are (a) the tetragonal T2 with Cr_5_B_3_ prototype, the same as D8_l_ αNb_5_Si_3_, where B substitutes Si and becomes Nb_5_(Si,B)_3_, and (b) the hexagonal silicide with Mn_5_Si_3_ prototype, which is the same for the D8_8_ γNb_5_Si_3_, i.e., the metastable 5-3 silicide in the Nb–Si binary [13], and the D8_8_ Ti_5_Si_3_ [14], and in which B occupies the interstitial site of Si and the silicide becomes Nb_5_Si_3_B_x_ [10]. The latter is referred to as the D8_8_ silicide.

According to the 1600 °C isothermal section of Nb–Si–B by Nowotny et al. [10], the B and Si concentrations in the T2 and D8_8_ silicides are in the ranges (at.%) 5.5 < B < 21 and 17 < Si < 33, and 5 < B < 16 and 27 < Si < 37.5, respectively, the T2 and D8_8_ have homogeneity range along the direction parallel to the Si-B binary [10,15] and a composition variation (≤2.5 and 5.5 at.%, respectively) along the direction parallel to the Nb–Si binary [10] and the Nb content of the D8_8_ is in the range 54 < Nb < 60. The D8_8_ has Si + B ≤ 42.5 at.% whereas the T2 has Si + B around 38 at.%. At 1600 °C, Sun et al. [11] calculated the maximum solubility of B in T2 to be 23.5 at.%, the B concentration in D8_8_ in the range 10 < B < 15 at.% along the Si–B direction and the Nb concentration in D8_8_ to be 55.6 ± 1 at.%. The latter is in good agreement with the average of 57 at.% [10] and gives Nb/(Si + B) about 1.3 in D8_8_ compared with 1.7 for the T2. The B solubility in the D8_8_ decreases with increasing temperature [15].

In this paper, we study the microstructure and properties of four RM(Nb)ICs based on Nb-24Ti-xSi-yB with additions of Al, Cr or Mo (x = 16, 17 or 18, y = 6, 7 or 8) that were selected so that they belong in area C in the Δχ versus δ, ΔH_mix_ versus Δχ, ΔH_mix_ versus VEC or VEC versus δ maps in [1,2,5]. The nominal composition of each alloy (at.%) was Nb-24Ti-16Si-5Cr-7B (alloy TT2), Nb-24Ti-16Si-5Al-7B (TT3), Nb-24Ti-18Si-5Al-5Cr-8B (TT4) and Nb-24Ti-17Si-3.5Al-5Cr-6B-2Mo (TT8), respectively (Table 1). These alloys are essentially based on specific KZ series alloys to which B has been added. In other words, the basis or reference alloys for TT2, TT3, TT4 and TT8, respectively are the alloys KZ4, KZ7, KZ5 [16] and JG3 [17] (for the nominal compositions of the reference alloys see Table A1 in Appendix A). Relevant data for the reference alloys are used to study how the addition of B in each alloy of this study affected microstructure and properties.

The structure of the paper is as follows: the description of experimental procedures is followed by the presentation of the results for the microstructures, density and hardness of the alloys. Preliminary results of the oxidation of the alloys are presented to show the effect of the addition of B on oxidation at 800 and 1200 °C. The discussion of the results includes their scrutiny from the perspective of NICE.

## 2. Experimental Section

Large button ingots (0.6 kg) of the alloys (Table 1) were prepared from high purity (min. 99.99 wt.%) Al, Cr, Mo, Nb, Si, Ti and B (99.5 wt.%) in an argon atmosphere using arc melting with a non-consumable tungsten electrode and a water-cooled copper crucible. Each alloy was melted five times. The alloys were heat-treated for 100 h at 1500 °C (1200 °C for TT2, see next section). Each alloy was wrapped in Ta foil and was heat-treated in a tube furnace under a continuous flow of Ti gettered argon followed by furnace cooling to room temperature. The microstructures of the alloys were characterised with XRD (Philips diffractometer, Hiltonbrooks Ltd., Crewe, UK, Cu radiation, solid specimens, JCPDS database) and microanalysis using EPMA (JEOL 8600 electron probe micro analyser, Tokyo, Japan, operating conditions 9 kV with a beam current of 30 nA [18]) equipped with WDX and EDX spectrometers, elemental standards and BN and TiN standards. The samples were prepared following standard metallographic procedures, mounted in Bakelite and ground using SiC grinding papers (320 to 2400 grit) and polishing with diamond DUR clothes (6 and 1 μm finish).

The density of the alloys was measured using Archimedes principle and a Sartorious electronic precision balance, Sartorius Lab Instruments GmbH & Co. KG, Göttingen, Germany, equipped with a density measurement kit. The isothermal oxidation of the as cast (AC) alloys was studied at 800 and 1200 °C using Stanton Redcroft thermo-balances (Thermal Scientific plc., Odessa, TX, USA) and specimens from the as cast alloys. The weight of each sample was measured at the start and end of each oxidation experiment. A Mitutoyo HM-101 hardness testing machine (Mitutoyo America, Aurora, IL, USA) with Vickers indenter was used to measure the hardness (0.5 kg load) of the as cast (AC) and heat-treated (HT) alloys. The microhardness of the solid solution and T2 silicide was measured in the AC and HT alloys using a 0.025 kg load. At least ten measurements were done for each alloy and condition, and each phase. The area fraction of the solid solution was measured using the software KS Run 3 in a Polyvar Met microscope. The SEM images of the large areas (×250) of the as cast (AC) and heat-treated (HT) alloys that were analysed by EPMA were used for the measurements.

## 3. Results

The actual composition of each alloy in the AC or HT condition is given in Table 1. The densities of the alloys are given in Table 2. The alloys TT4 and TT2 had the lowest and highest density, respectively, and the densities of all four alloys were lower than 7 g/cm^3^. The macrosegregation of solutes (MACX, X = Al, B, Cr, Si, Ti) in the alloys is given in Table 3. Note that this table has data for the Al and Cr free alloy TT1, to help us understand the role of boron with other alloying additions on MACX. The highest macrosegregation of B and Si was observed respectively in the alloys TT8 and TT4 whereas the macrosegregation of Ti was essentially similar in all four alloys. The phases that were observed in the alloys using XRD and EPMA are summarised in Table 4. The chemical compositions of the phases that were observed in all parts of the ingots are given in Table 5.

### 3.1. As Cast Alloys

**Figure 1:**
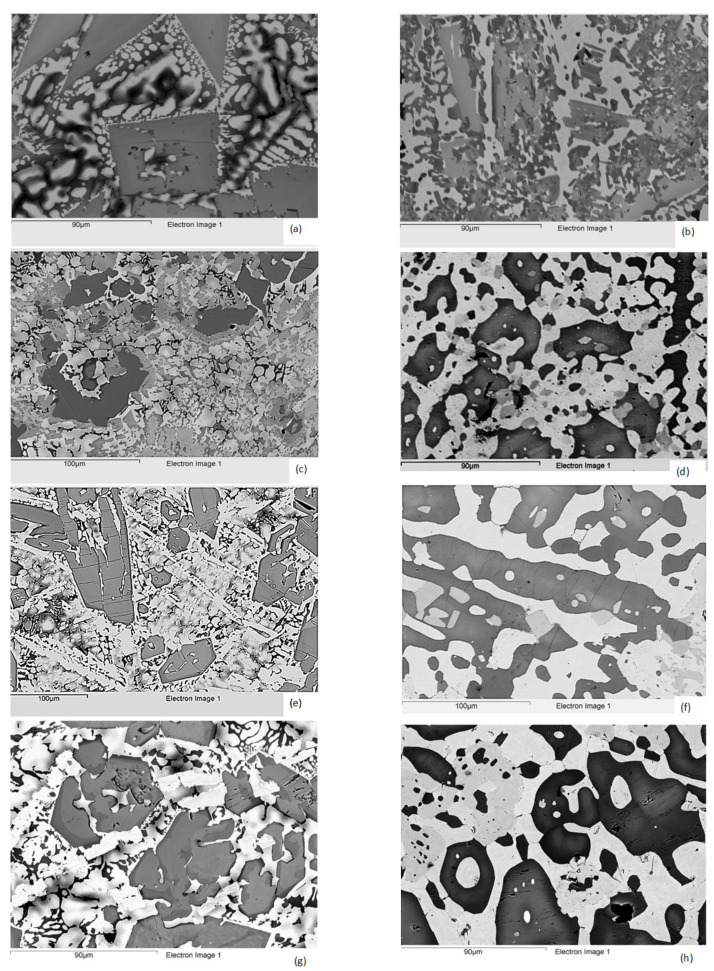
Microstructures of the AC (**a**,**c**,**e**,**g**) and HT (**b**,**d**,**f**,**h**) alloys TT2 (**a**,**b**), TT3 (**c**,**d**), TT4 (**e**,**f**) and TT8 (**g**,**h**). Note that contrast enhancement has been applied to show different phases. For description of microstructures see text.

In all parts of the ingot of the alloy TT2, were observed the Nb_ss_, T2, a Cr and Ti-rich phase and a eutectic of the Nb_ss_ and T2, whereas in specific parts of the ingot other phases were also present, namely a Ti-rich phase in the top and bottom, a C14-Laves phase in the top and bulk and Nb_3_Si silicide in the bulk (Table 4 and Table 5, Figure 1a and Figure S1a in Supplementary Materials). Ti-rich Nb_ss_ and Ti-rich T2 were also observed. In Figure 1a, the grey contrast facetted phase is the T2, the bright contrast phase is the Nb_ss_, darker contrast areas at the edges of Nb_ss_ are Ti-rich Nbss and darker grey contrast areas in between Nb_ss_ are Ti-rich T2. The Ti-rich phase, C14-Laves phase, Cr and Ti-rich phase and Nb_3_Si were observed in the dark and very contrast inter-dendritic Nb_ss_ areas. The average compositions of the Ti-rich phase, the Laves phase and the Nb_3_Si silicide, respectively, were 23.2(18.6–27.9)Nb-52.3(47.8–56.7)Ti-8.9(8.4–9.4)Si-14.7(14–15.5)Cr-0.9(0.8–1)B, 13.4(12.6–14.1)Nb-31.6(27.5–34.2)Ti-7.8(6.7–9)Si-47.7(45.3–49)Cr and 25.9(21.7–29)Nb-42.4(43–44.1)Ti-23.4(19.4–26.5)Si-7.9(4–11.3)Cr, where in the parentheses are given the minimum and maximum analysis values for each phase. It should be noted that the Laves and Nb_3_Si compounds were B free and that the Si concentration in the silicide would suggest also the presence of metastable Nb_3_Si [11], which however was not indicated by the XRD (Figure S1a in Supplementary Materials). In the Nb_ss_, with increasing Ti concentration, the concentrations of B and Cr respectively decreased and increased, whereas in the T2 the B concentration decreased with increasing Ti concentration (Table 5). The average <Si> content and the <Nb>/<Si> and Si/B ratios of the T2 and Ti-rich T2 were 38.2 and 37.1 at.%, 1.6 and 1.7, and 2 and 3.8, respectively (where <Nb> = Nb + Cr + Ti and <Si> = B + Si). The T2 silicide was facetted, this was particularly noticeable in the top and bottom of the ingot. The vol.% of the eutectic was lower in the bulk.

In all parts of the ingot, the microstructure of the alloy TT3 consisted of the Nb_ss_, T2, and eutectic of these two phases (Table 4 and Table 5, Figure 1c and Figure S1c in Supplementary Materials). The XRD suggested the presence of the D8_8_ silicide, however, its presence was not confirmed by EPMA. Ti-rich Nb_ss_ and T2 were also observed but in this alloy the partitioning of Ti to T2 was very strong and resulted in distinct separate T2 grains very rich in Ti (and Al) in the top and bulk of the ingot, with average composition 34.2(29.8–37.2)Nb-28.2(24.7–32.2)Ti-27.8(26.6–28.8)Si-4.2(3.7–4.9)Al-5.6(3.6–7.4)B. In Figure 1c, the light contrast phase is the Nb_ss_, the grey contrast phase is the T2 and the darker grey contrast is the Ti-rich T2. In the Nb_ss_ and T2, with increasing Ti concentration, the concentrations of both B and Al decreased in the solid solution, and respectively decreased and increased in the T2. The average <Si> content, and the <Nb>/<Si> and Si/B ratios of the T2, Ti-rich T2 and T2 very rich in Ti were 38.6, 39 and 37.5 at.%, 1.6, 1.56 and 1.67, and 1.9, 4 and 5, respectively (where <Nb> = Nb + Ti and <Si> = Al + B + Si). The T2 was facetted only in the bulk of the ingot. Cracks that had formed in large Ti-rich T2 grains were stopped by the surrounding solid solution.

In all parts of the ingot of the alloy TT4, the Nb_ss_, T2, D8_8_ and eutectic of Nb_ss_ and T2 were observed. The D8_8_ silicide exhibited very bright contrast (Table 4 and Table 5, Figure 1e and Figure S1e in Supplementary Materials). Ti-rich Nb_ss_ and Ti-rich T2 were also observed but there was no evidence of Ti-rich D8_8_. The latter was Al free. In Figure 1e, the T2 is the grey contrast phase, darker grey contrast areas correspond to Ti-rich T2, the very bright contrast phase with no darker contrast areas is the D8_8_ (e.g., the very bright contrast thin long phase at the bottom left-hand side, middle and upper right-hand side of the image “running” at about 45 degrees from right to left) and the bright phase is the Nb_ss_ with darker areas corresponding to the Ti-rich Nb_ss_.

In the Nb_ss_ and T2, with increasing Ti concentration, the concentrations of B and Cr decreased and increased, respectively, and the concentration of Al essentially did not change in the solid solution, whereas in the T2 the Al and Cr increased and the B decreased. The average <Si> content, and the <Nb>/<Si> and Si/B ratios of the T2, Ti-rich T2 and D8_8_ were 38.2, 36.7 and 40.8 at.%, 1.62, 1.72 and 1.45, and 4.3, 6.4 and 0.6, respectively (where <Nb> = Nb + Ti + Cr and <Si> = Al + B + Si). The T2 was facetted in the top and the bulk of the ingot and Ti-rich T2 grains were cracked with the cracks parallel to each other and not extending in the surrounding Nb_ss_.

The microstructure of the alloy TT8 consisted of Nb_ss_, T2, D8_8_, Nb_ss_ + T2 eutectic in all parts of the ingot and Nb_3_Si that was observed only in the top and bulk of the ingot (Table 4 and Table 5, Figure 1g and Figure S1g in Supplementary Materials). The average composition of Nb_3_Si was 46Nb-23Ti-20.1Si-2.8Al-10Cr-5.8B-1.3Mo. The D8_8_ exhibited very bright contrast and was Al free. In Figure 1g, the T2 is the grey contrast phase, darker grey contrast areas correspond to Ti-rich T2, the very bright contrast phase with no darker contrast areas around it is the D8_8_, and the bright phase with darker areas is the Nb_ss_. The Nb_3_Si exhibits very dark contrast in the inter-dendritic Nb_ss_ areas. The concentration of B in the solid solution varied significantly. It should be noted that there were solid solution grains that were B free. Ti-rich Nb_ss_ and Ti-rich T2 were also observed, and no Ti-rich D8_8_. In the Nb_ss_ and T2, with increasing Ti concentration, the concentrations of Al, B and Mo decreased and that of Cr increased in the solid solution whereas in the T2 the B decreased but the changes of Al, Cr and Mo were minimal. The average <Si> content, and the <Nb>/<Si> and Si/B ratios of the T2, Ti-rich T2 and D8_8_ were 38.9, 37.7 and 42.6 at.%, 1.57, 1.65 and 1.35, and 2.5, 3.3 and 0.4, respectively (where <Nb> = Nb + Ti + Cr and <Si> = Al + B + Si). Both the T2 and D8_8_ were facetted, particularly the latter and there were cracks in T2 that run parallel to each other and stopped at the Nb_ss_.

The vol.% of the Nb_ss_ was essentially the same in the alloys TT2, TT3 and TT8, about 38% and significantly lower in the alloy TT4 (Table 2). The microhardness of the solid solution was lowest and highest respectively in the alloys TT2 and TT4 (472 and 563 HV, respectively), the microhardness of T2 was highest and lowest in the alloys TT2 and TT3, respectively (1346 and 1232 HV, respectively), and the highest and lowest alloy hardness was measured for the alloys TT8 and TT3, respectively (744 and 630 HV, respectively), see Table 2.

### 3.2. Heat Treated Alloys

All the alloys were heat-treated at 1500 °C with the exception of the alloy TT2 that exhibited liquation at this temperature and was heat-treated at 1200 °C. The phases that were present in their microstructures are given in Table 4. The alloys TT2 and TT8 were contaminated by nitrogen during the heat treatment.

In the alloy TT2 the Nb_3_Si silicide, the Ti-rich phase, the Cr and Ti-rich phase and the Laves phase that were present in the cast alloy were not observed. The Nb_ss_ and T2 were present with Ti-rich areas in the latter (Table 4 and Table 5, Figure 1b and Figure S1b in Supplementary Materials). In Figure 1b, the light contrast areas are the Nb_ss_, the grey contrast phase is the T2, the darker grey contrast is the Ti-rich T2 and the very dark areas are TiN. The chemical inhomogeneity that was characteristic of this alloy in the as cast condition resulted in T2 grains in the heat-treated microstructure with <Nb>/<Si> and Si/B ratios covering a wider range than in the other alloys. The chemical composition of T2 and Ti-rich T2 did not change significantly compared with the cast alloy (Table 5), the former had <Si> = 37.2 at.% (<Nb>/<Si> = 1.69) and Si/B about 1.9 whereas the latter had Si + B in the range 34.1 to 37 at.% (or <Nb>/<Si> in the range 1.7 to 1.93) and Si/B about one. The average composition of the Ti-rich T2 was as follows in different parts of the heat-treated ingot: in the top 39.9(37.7–42.4)Nb-20.8(17.4–24.1)Ti-18.6(17.1–19.6)Si-2.2(1.8–2.9)Cr-18.5(16.7–20.2)B, in the bulk 42.4(41.4–43.9)Nb-20.6(19.4–21.2)Ti-19.7(18.4–21.4)Si-2.2(1.7–2.7)Cr-15.2(13.3–17.6)B, and in the bottom (where only one analysis was possible) 41.5Nb-19.2Ti-18.1Si-2.4Cr-18.9B. In the bottom of the heat-treated alloy there were also areas of the Ti-rich T2 where the Si and B concentrations were not equal, with <Si> = 37 at.% (<Nb>/<Si> = 1.7) and Si/B about 3.8 (41.7(39.6–43.5)Nb-20.8(18.4–22.4)Ti-29.3(27.9–30.3)Si-7.7(6.5–9.8)B-0.5(0.4–0.7)Cr). Furthermore, there were Ti-rich T2 grains with <Si> = 35.1 at.% (<Nb>/<Si> = 1.85) and Si/B = 6.3 (where <Si> = Si +B, <Nb> = Nb + Cr + Ti).

The microstructures of the alloys TT3 and TT4 consisted of the Nb_ss_, T2 with the D8_8_ present only in the latter alloy and no TiN in both alloys. Ti-rich T2 was observed in TT3 but not in TT4 and the D8_8_ was Al free, as was the case in the cast alloy (Table 4 and Table 5, Figure 1d,f and Figure S1d,f in Supplementary Materials). The microstructure of TT3-HT is shown in Figure 1d, where the light contrast phase is the Nb_ss_, the grey contrast phase is the T2 and the darker grey contrast is the Ti-rich T2. In the alloy TT3, the average <Si> content, and the <Nb>/<Si> and Si/B ratios of the T2 and Ti-rich T2 were 38.5 and 37.7 at.%, 1.6 and 1.65, and 2.6 and 6.1, respectively (where <Nb> = Nb + Ti and <Si> = Al + B + Si). In the TT4-HT there were no facetted T2 grains and often the D8_8_ silicide was observed inside (surrounded by) T2 grains. The microstructure of TT4-HT is shown in Figure 1f, where the bright phase is the Nb_ss_, the grey phase is the D8_8_ and the darker grey contrast phase is the T2. The T2 had <Si> = 39 at.%, <Nb>/<Si> = 1.56, Si/B = 5.5 (<Si> = Al + B + Si, <Nb> = Nb + Cr + Ti), whereas the D8_8_ had <Si> = 42.7 at.%, <Nb>/<Si> = 1.3 and Si/B = 0.5. In the alloy TT8 the microstructure consisted of Nb_ss_, the T2, D8_8_ and Ti-rich T2 silicides, was contaminated by nitrogen and the Nb_3_Si was not present (Table 4 and Table 5, Figure 1h and Figure S1h in Supplementary Materials). The D8_8_ was Al free. The microstructure of TT8-HT is shown in Figure 1h, where the bright phase is the Nb_ss_, the grey phase is the D8_8_, the darker grey contrast phase is the T2 and dark areas around the latter correspond to Ti-rich T2. The average <Si> content, and the <Nb>/<Si> and Si/B ratios of the T2, Ti-rich T2 and D8_8_ were 37.3, 38.3 and 40 at.%, 1.68, 1.61 and 1.5, and 6.2, 4.1 and 0.51, respectively (<Nb> = Nb + Cr + Mo + Ti). Some grains for the Nb_ss_ were B free, as in TT8-AC.

After the heat treatment, the vol.% of Nb_ss_ increased slightly in the alloys TT2, TT3 and TT8 and significantly in TT4 and was similar in all alloys, about 39% (Table 2). The microhardness of the Nb_ss_ was reduced in all alloys, most noticeably in TT3. Similarly, the microhardness of T2 was reduced in all alloys, but the smallest reduction was in TT4. The hardness of all alloys was reduced, the TT8 still had the highest hardness but the smallest hardness reduction was measured for TT2 (Table 2).

### 3.3. Oxidation

**Figure 2:**
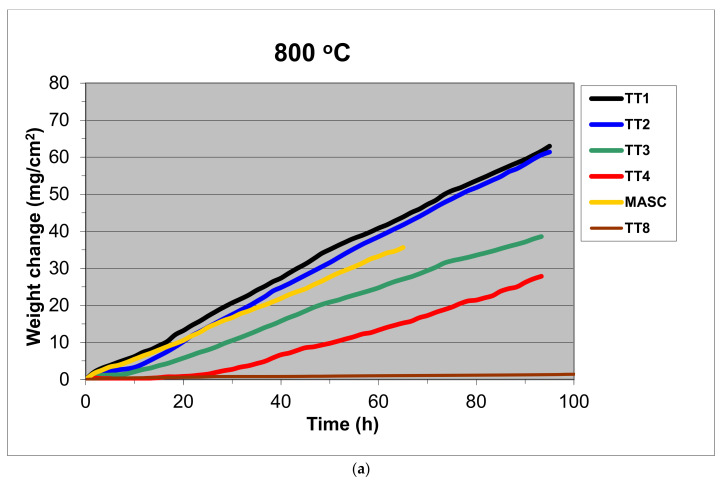

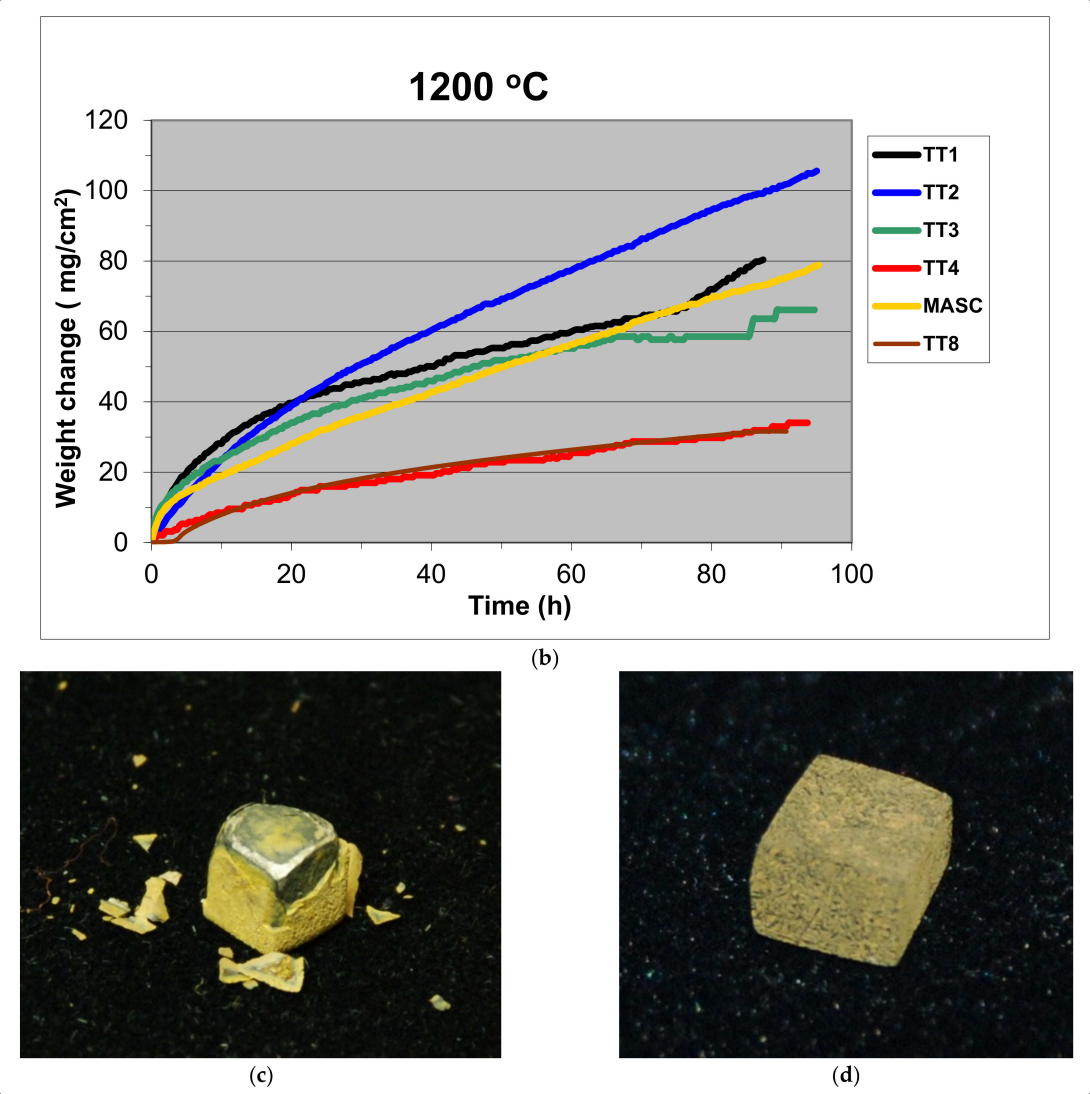
Mass change (ΔW/A) versus time of alloys TT1, TT2, TT3, TTT4, TT8 and MASC at (**a**) 800 °C and (**b**) 1200 °C. Colours: black TT1, blue TT2, green TT3, red TT4, yellow MASC, brown TT8. Specimens of TT4 and TT8 after oxidation (**c**) at 1200 °C and (**d**) 800 °C, respectively.

The mass change data is shown in Figure 2a,b for isothermal oxidation at 800 and 1200 °C and typical examples of scale spallation at the two temperatures are shown in Figure 2c,d. The Figure 2a,b include data for the MASC alloy (Nb-25Ti-16Si-2Al-2Cr-8Hf [20]) and the alloy TT1 [12]. The oxidation rate constants are given in Table 6. At 800 and 1200 °C, all four alloys followed respectively linear and parabolic oxidation kinetics. None of the alloys with B addition suffered from pest oxidation at 800 °C, meaning each alloy did not convert into powder, but the scales that formed on the alloys TT1, TT2, TT3 and TT4 spalled off. At 1200 °C scale spallation was also observed for the alloys TT1, TT2, TT3 and TT4, and the MASC alloy. The 1200 °C specimen of TT8 looked exactly like the specimen shown in Figure 2d.

## 4. Discussion

### 4.1. Macrosegregation

The addition of B increased the macrosegregation of Ti and Si compared with the reference B free alloys, with the exception of TT8 where MACSi decreased compared with JG3 (Table 7). TT4 had the highest MACSi, an increase of 3.4 at.% compared with KZ5, and TT1 had the highest MACTi. The highest and lowest MACB was exhibited respectively by TT8 and TT4. With the exception of the alloy TT4, the spread of MACB was narrow and around 4.4 at.%.

**Figure 3:**
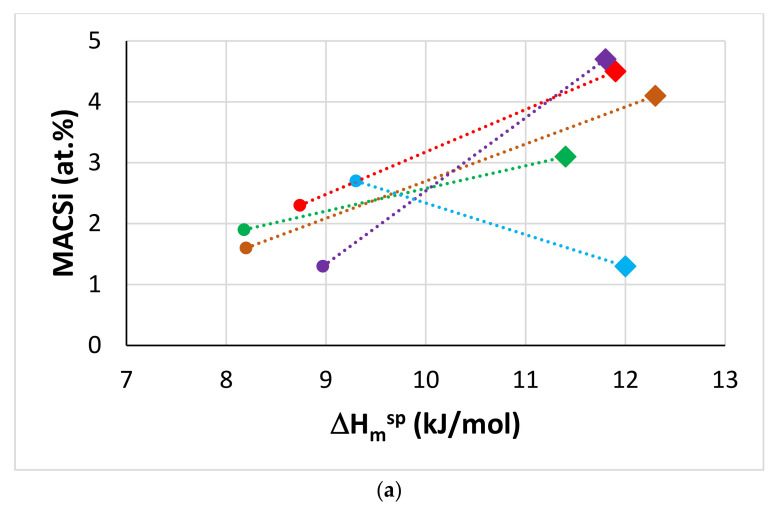

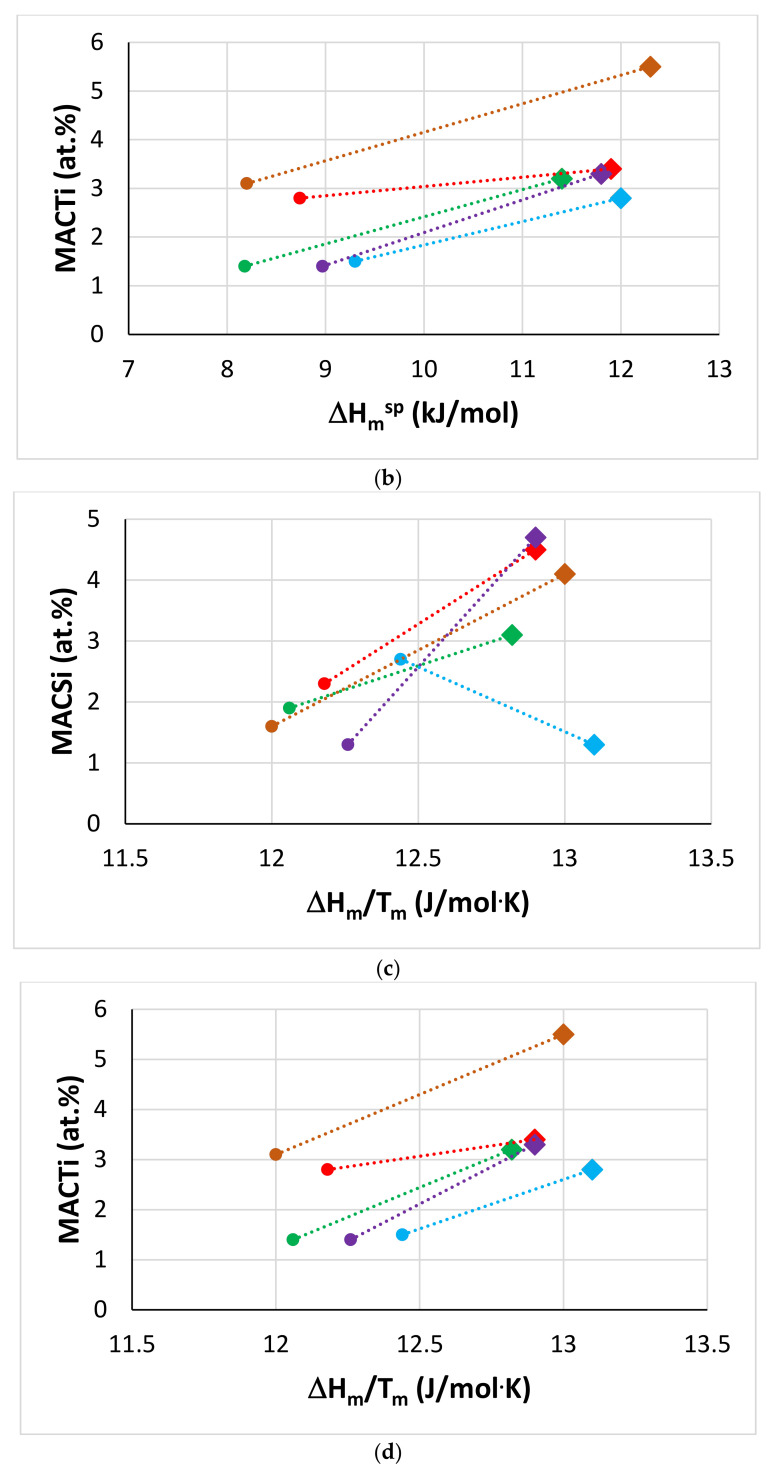

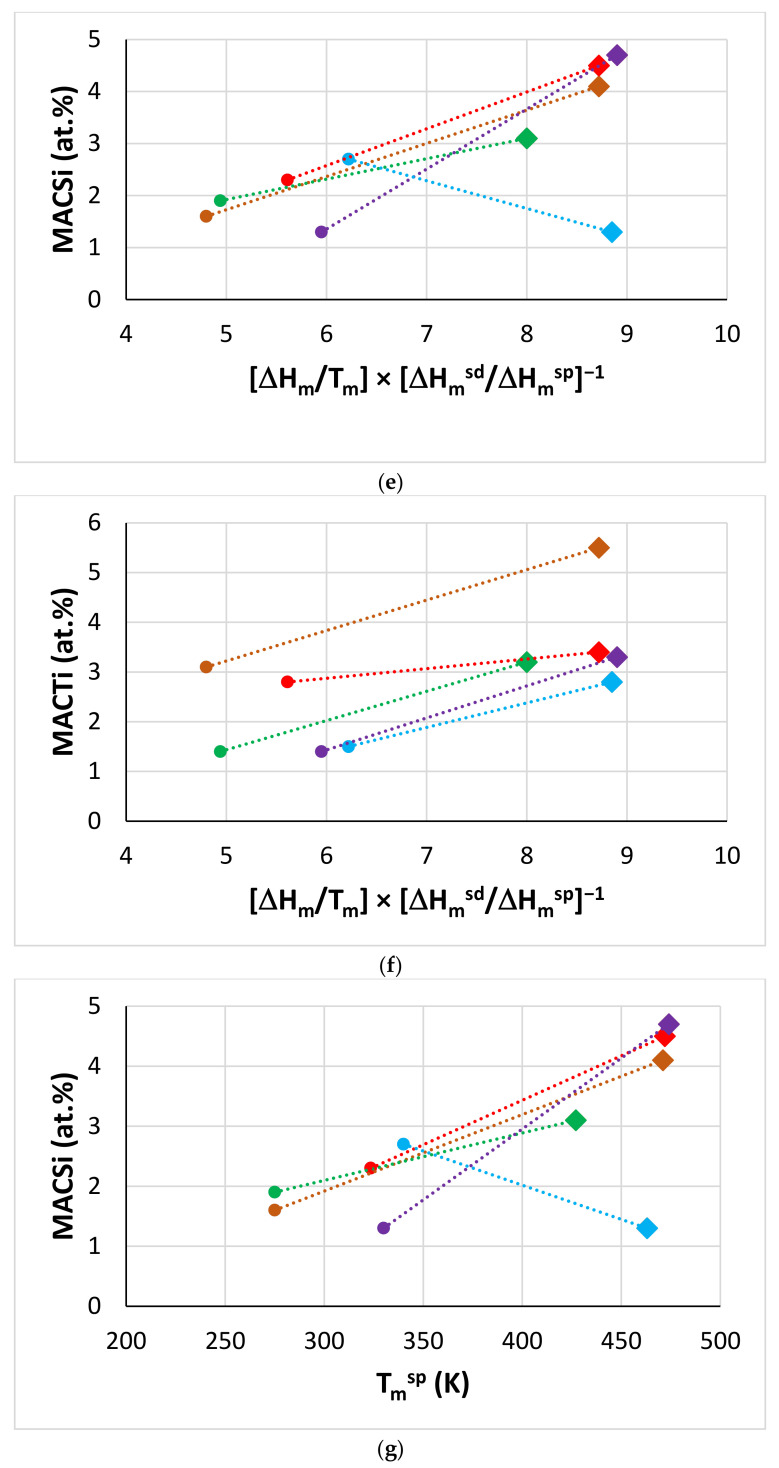

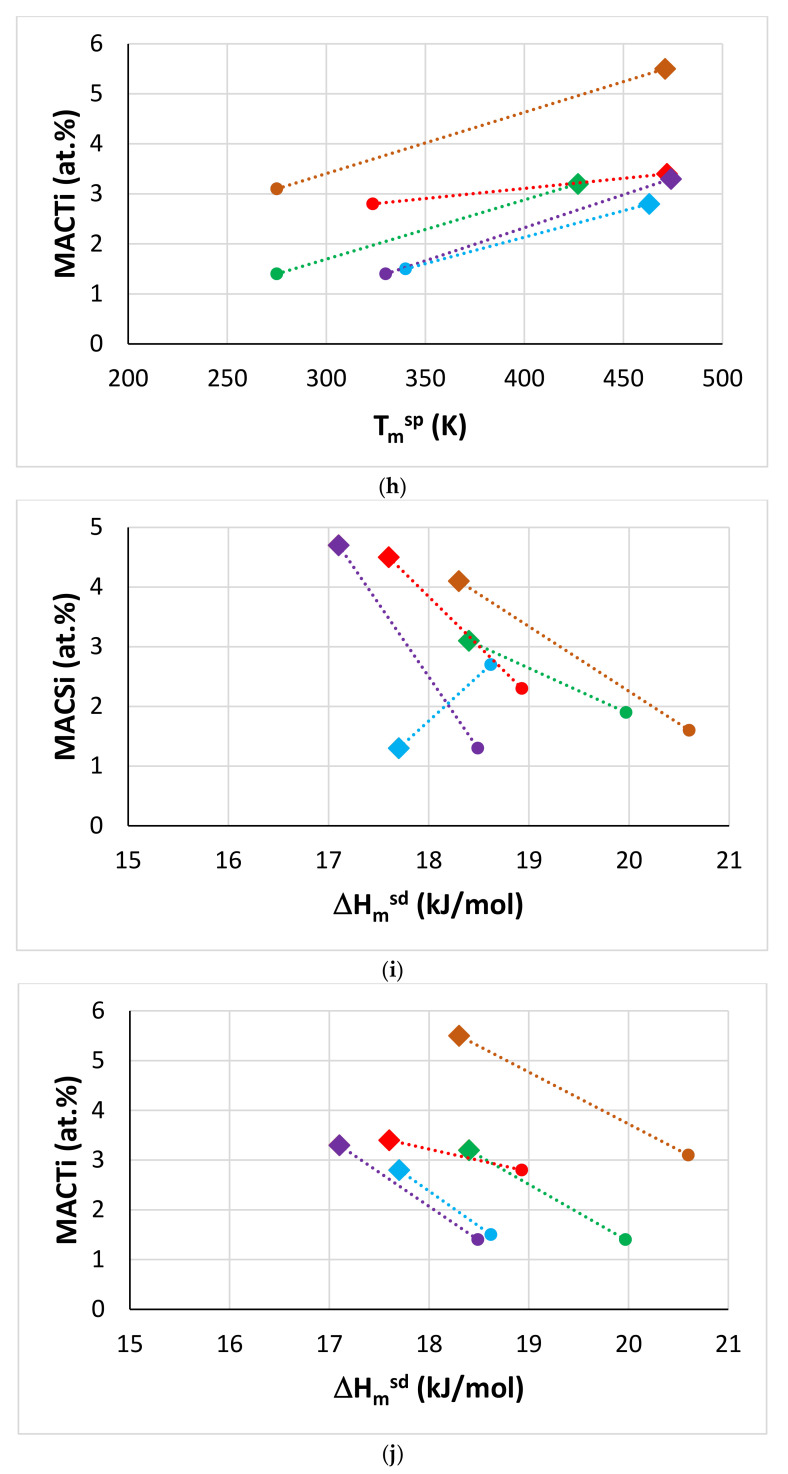

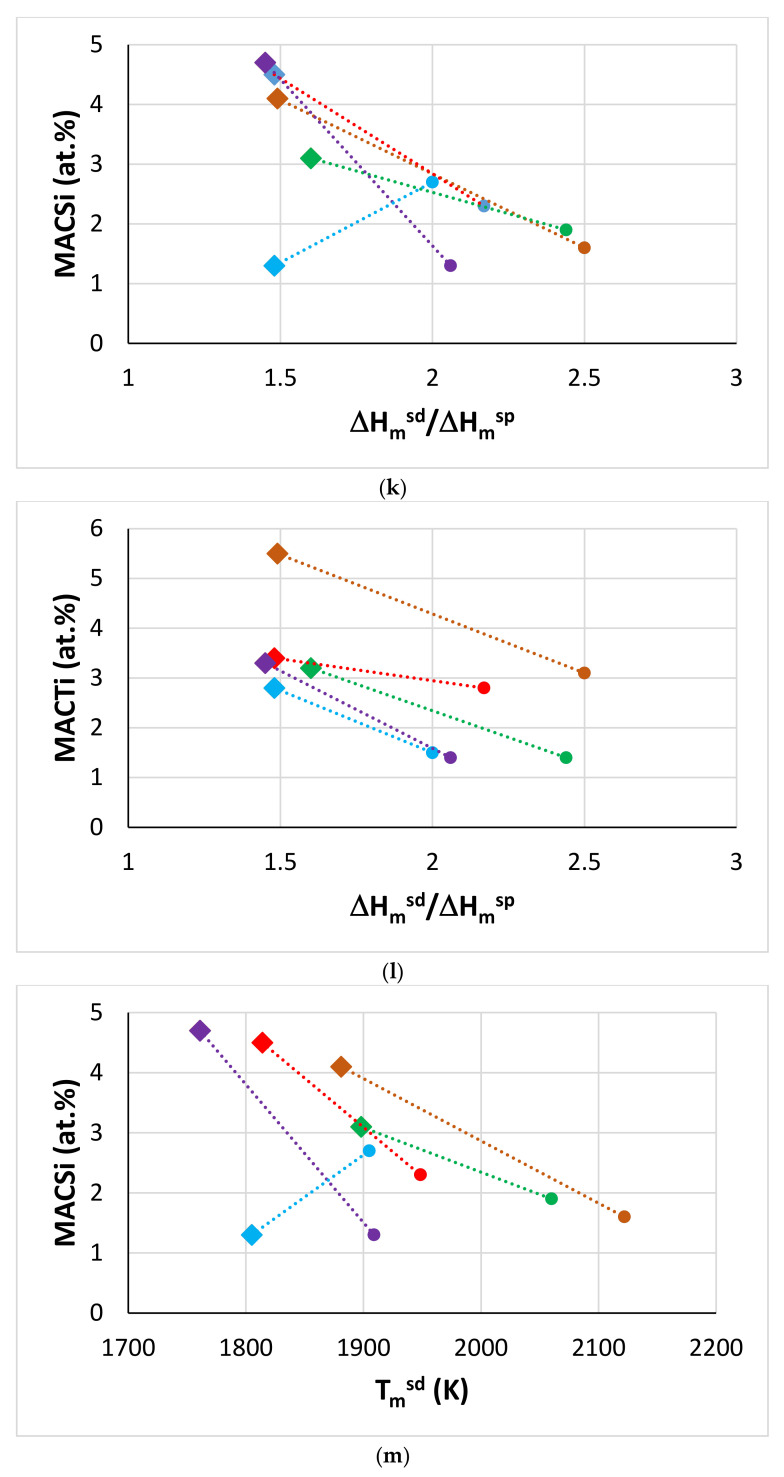

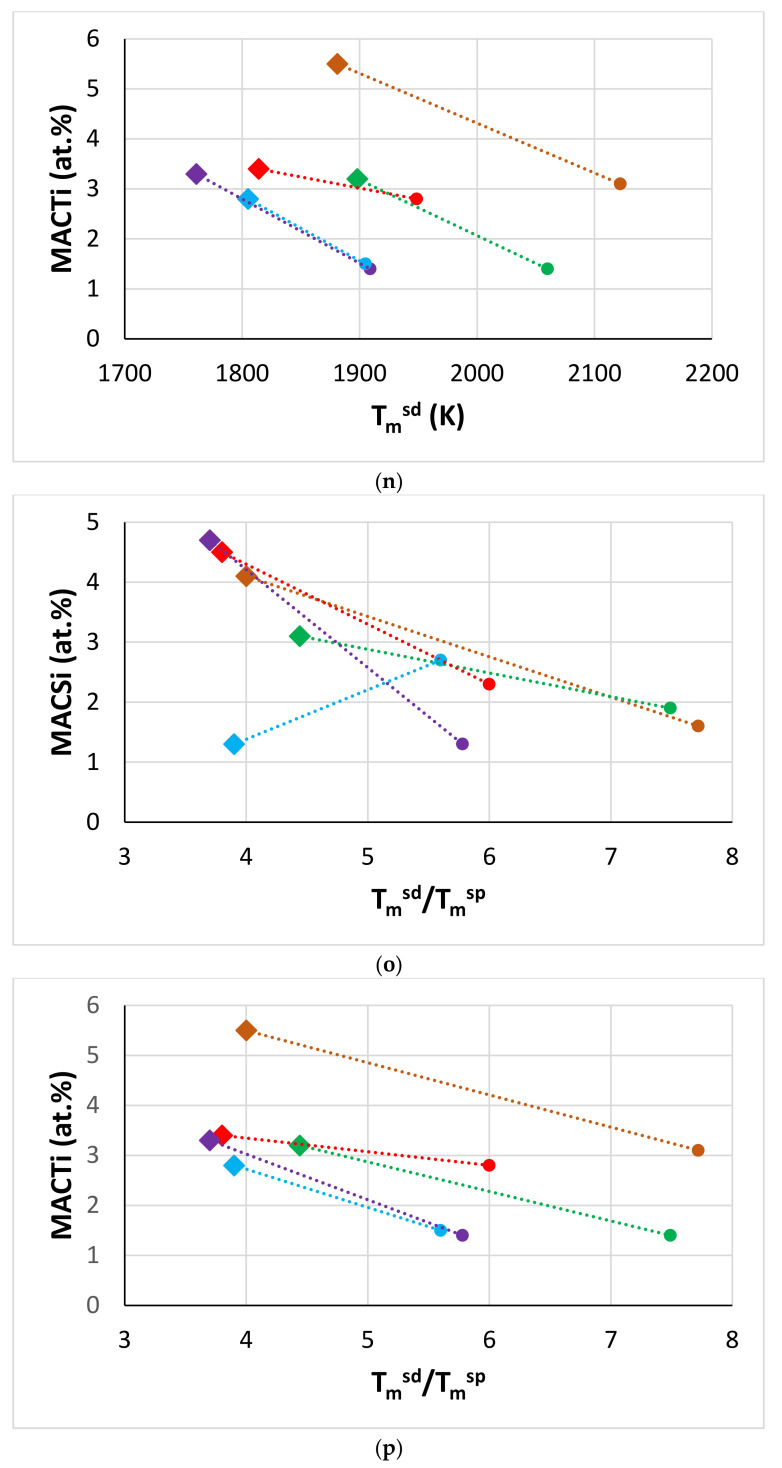

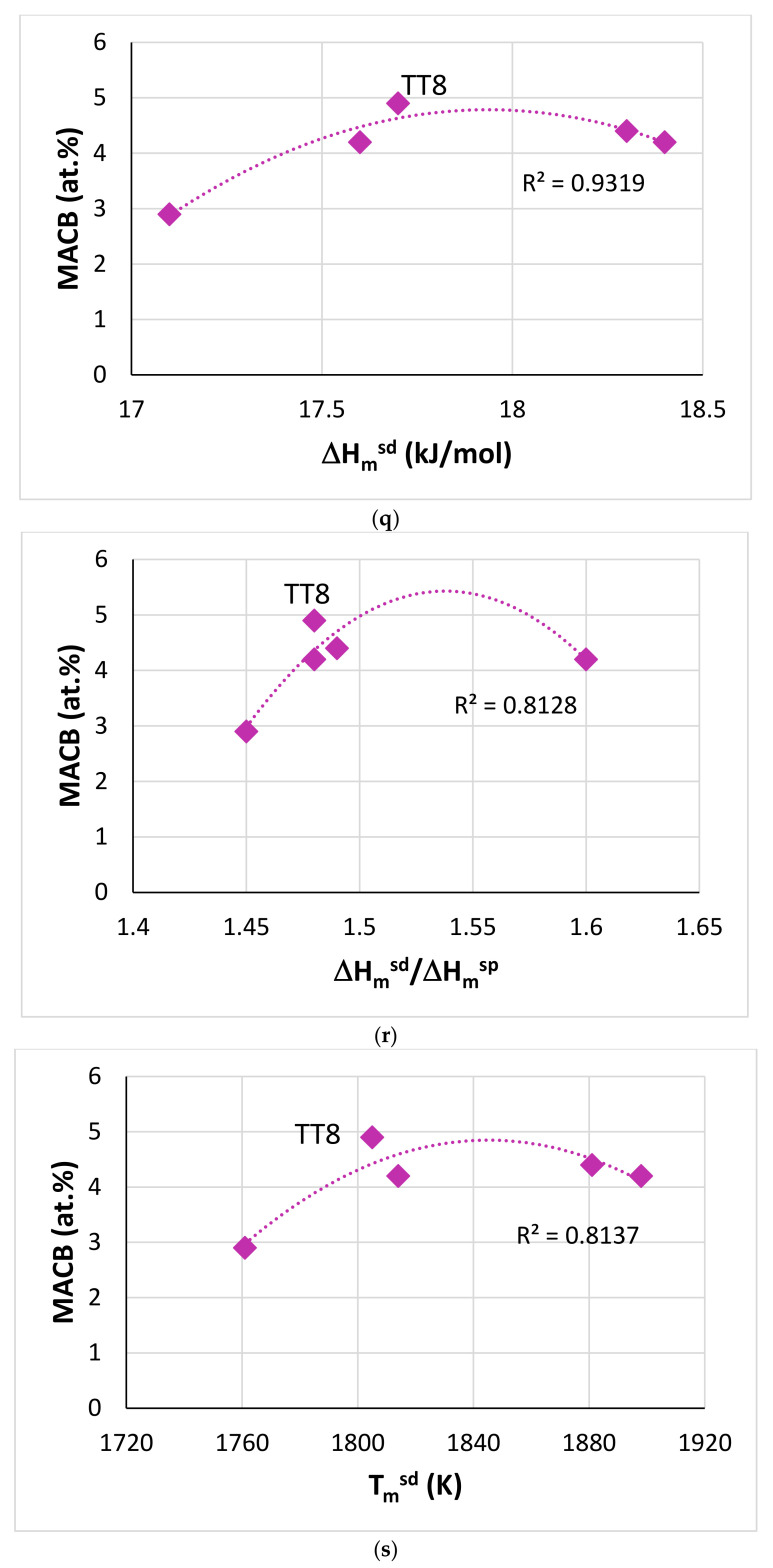
MACSi (**a**,**c**,**e**,**g**,**i**,**k**,**m**,**o**), MACTi (**b**,**d**,**f**,**h**,**j**,**l**,**n**,**p**) and MACB (**q**,**r**,**s**) versus parameters for alloys TT1, TT2, TT3, TT4 and TT8, and their basis/reference alloys KZ3, KZ4, KZ5, KZ7 and JG3. Diamonds for B containing alloys. Colours (**a**–**p**), brown TT1, KZ3, green TT2, KZ4, red TT3, KZ7, purple TT4, KZ5, blue TT8, JG3. Pink for the MACB data. The alloy TT8 is indicated in (**q**) to (**s**). If TT8 were to be excluded, the R^2^ values for the parabolic fit of the data change to 0.9986, 0.813 and 1, respectively in (**q**,**r**,**s**).

The macrosegregation of Si and Ti in the alloys of this study and their reference alloys is shown in Figure 3a–p. This figure shows plots of MACSi and MACTi versus specific parameters that describe macrosegregation in RM(Nb)ICs [19]. In agreement with previous research on B free RM(Nb)ICs [21,22,23,24], RM(Nb)ICs/RCCAs [25] and HEAs [26] both MACSi and MACTi increased with ΔH_m_^sp^, ΔH_m_/T_m_, [ΔH_m_/T_m_] × [ΔH_m_^sd^/ΔH_m_^sp^]^−1^ and T_m_^sp^, and decreased with ΔH_m_^sd^, ΔH_m_^sd^/ΔH_m_^sp^, T_m_^sd^ and T_m_^sd^/T_m_^sp^ (for definitions and equations for above parameters, please refer to [19]). This means that alloy design in NICE can optimise both MACSi and MACTi in B containing alloys using the same parameters. It should be noted that in the MACSi plots the data for the alloy TT8 and its basis JG3 does not follow the aforementioned trend. Further research is essential to confirm MACSi in TT8 and to study RC(Nb)ICs based on TT4 with RM addition(s) as both alloys (i.e., TT4 and TT8) point to a “pathway” for developing RCCAs with B addition [1,2].

The data for MACB is limited only to the alloys of this study and the alloy TT1 [12]. Figure 3q to s show a good parabolic fit of this limited data of MACB versus the parameters ΔH_m_^sd^, ΔH_m_^sd^/ΔH_m_^sp^ and T_m_^sd^, and suggests “maximum” MACB between five and six at.% for ΔH_m_^sd^ ≈ 18 kJ/mol, ΔH_m_^sd^ ≈ 1.535 kJ/mol and T_m_^sd^ ≈ 1840 K. More data about MACB is needed before we can firmly ascertain that the same parameters and trends also describe the macrosegregation of B in RM(Nb)ICs, and RHEAs or RCCAs with B addition.

### 4.2. Microstructures

The alloy microstructures were chemically inhomogeneous, particularly in the case of the alloy TT2. In the reference alloys KZ7, KZ5 and JG3, the high-temperature tetragonal βNb_5_Si_3_ had formed from the melt [16,17], whereas with the addition of B, in the alloys TT2, TT3, TT4 and TT8 the tetragonal T2 silicide (same structure as the low-temperature αNb_5_Si_3_, see introduction) formed from the melt as did the hexagonal D8_8_ silicide in the alloys TT4 and TT8. In the reference alloy KZ4, the αNb_5_Si_3_ formed via the eutectoid transformation of Nb_3_Si [16]. Solute partitioning made the identification of phases, in particular the D8_8_ silicide in the alloys TT4 and TT8, difficult. In all four alloys, the Nb_ss_ and T2 were stable phases and the D8_8_ in TT4 and TT8.

In the alloy TT2 the Ti-rich phase, Nb_3_Si and C14 Laves phase formed in competition with the Cr and Ti-rich phase (Table 4) in specific parts of the ingot of the cast alloy. The Ti-rich phase was observed only in the top and bottom of the ingot, the Laves phase in the top and bulk, similarly to KZ4-AC [16], and the Nb_3_Si silicide only in the bulk. The aforementioned four phases were not stable in the heat-treated alloy. Note that the C14 Laves phase was also not stable in KZ4-HT. The Nb_ss_ + Nb_3_Si eutectic that formed in KZ4-AC was replaced by the Nb_ss_ + T2 eutectic in TT2-AC. In the latter, the area fraction of the eutectic was significantly reduced in the bulk of the ingot rather than at the bottom, which was the case in TT1-AC [12], and would suggest sensitivity of the Nb_ss_ + T2 eutectic to cooling rate and the presence or not of the Nb_3_Si silicide. The Laves phase formed from Cr rich melt and thus depended on the partitioning of Cr during solidification. This would explain why the Laves phase was not observed in the bottom of the ingot. Compared with KZ4-AC, no Ti-rich areas were observed in the Laves phase in TT2-AC and the Ti concentration was significantly higher than in the Laves phase in KZ4-AC. This was attributed to the Laves phase “competing for Ti” with the Cr and Ti-rich phase (which formed everywhere in the ingot) and the Ti-rich phase (which formed in the top and bottom of the ingot). Chemical inhomogeneity existed in TT2-HT, with a range of composition of the Ti-rich T2 (see Table A2 in the Appendix A) indicating that equilibrium was not reached in this alloy after 100 h at 1200 °C.

The as solidified microstructure in TT3 was similar to that in KZ7-AC [16], the Nb_ss_ + βNb_5_Si_3_ eutectic in the latter was replaced by the Nb_ss_ + T2 eutectic in TT3-AC. Compared with TT1-AC, the addition of Al in TT3 suppressed the formation of Nb_3_Si, which was also the case in KZ7-AC compared with KZ3-AC [16]. The suppression of the Nb_3_Si was also linked with the strong partitioning of Ti in the T2 that “starved” the melt of Ti, which stabilises the Nb_3_Si. Therefore, the synergy of B and Al in TT3 furthered the formation of primary tetragonal T2 (as did the addition of Al in KZ7 regarding the formation of primary βNb_5_Si_3_ [16]) and of the Nb_ss_ + T2 eutectic. The partitioning of Ti in T2 was sensitive to cooling rate as the very Ti-rich T2 was not observed in the bottom of the ingot of TT3-AC.

In the alloy TT4, the C14 Laves phase was not formed, differently with KZ5-AC [16]. In the alloy TT8-AC, the Nb_3_Si silicide was formed but not the C14 Laves, similarly with JG3-AC [17], in which Nb_ss_ + Nb_3_Si eutectic was formed compared with the Nb_ss_ + T2 eutectic in TT8-AC. The Nb_3_Si was not observed in the bottom of the ingot of TT8-AC. Comparison with the data for JG3-AC [17] and TT8-AC would suggest sensitivity of the formation of Nb_3_Si to cooling rate that was attributed to the synergy of B, Cr and Mo in which B and Cr lead. Three-phase equilibria between Nb_ss_, Nb_5_Si_3_ and Nb_4_Si (metastable silicide) would exist for an alloy of composition Nb-17.5Si-2Mo (at.%) according to the ternary Nb-Si-Mo system by Savitskiym et al. [27]. The Nb_3_Si in TT8-AC had Si + B + Al ≈ 28.7 at.%, which is close to the composition of the metastable Nb_3_Si-m in [13], and the Si content (20.1 at.%) corresponds to Nb_4_Si (metastable silicide, [13]). Thus, it is likely that with the addition of Mo both the stable (i.e., tetragonal) and metastable Nb_3_Si silicide(s) formed in TT8-AC. Compared with JG3-AC, the Nb_3_Si was leaner in Si and Al and richer in Cr; this was attributed to the synergy of Mo and B in TT8. The concentration of Mo was the same in the Nb_3_Si in JG3-AC and TT8-AC, which would suggest that B did not affect the concentration of Mo in the 3-1 silicide. Compared with TT1-AC [12], the Nb_3_Si in TT8-AC was leaner in Si but richer in B. Compared with TT2-AC, the Nb_3_Si in TT8-AC was leaner in Si but richer in Cr and B. Given that in TT2-AC the Nb_3_Si was B free, it was concluded that in the presence of Mo the solubility of B in Nb_3_Si is increased and that Mo leads in the Mo and Cr synergy and eliminates the negative effect of Cr on the solubility of B in Nb_3_Si.

In both the TT4 and TT8 alloys (Ti/Si = 1.5 based on nominal compositions or average Ti/Si = 1.42 based on actual chemical compositions, Table 1), with the addition of B, a new B rich phase formed and was stable, namely the D8_8_ silicide. The latter was observed in the areas surrounding the T2 and exhibited very strong bright contrast, which was stronger than that of Nb_ss_ (Figure 1e,g). There was no solubility of Al in D8_8_ and its Ti concentration was in the range 10.2 < Ti < 12.5 at.% (average 11.7 at.%, Table 5). In all the alloys of this study, whether in the AC or HT condition, the Si + B or the Si + B + Al content of the T2 was essentially the same (37 and 38.1 at.%, respectively, average 37.6 at.%) whereas the Si + B content of the D8_8_ was higher and in the range 40 to 42.7 at.% (average 41.5 at.%). Furthermore, the Si/B ratio in the T2 was in the range 1 to 6.4 (average 4) compared with 0.5 for the D8_8_ silicide (range 0.4 to 0.6), and the average <Nb>/<Si> ratio was 1.7 in the T2 compared with 1.4 in the D8_8_ (Table A2 in Appendix A). The Ti concentration of the T2 and D8_8_, respectively, was in the ranges 14.3 to 30.7 at.% (average 21.2 at.%) and 10.2 to 12.5 at.% (average 11.7 at.%), Table 5. The above data for the T2 and D8_8_ silicides is in agreement with the Nb-Si-B ternary [10], see introduction.

In the 1600 °C ternary Nb-Si-B section, tentative equilibrium between T2 and Nb_3_B_2_ was indicated by Nowotny et al. [10]. Thermodynamic modelling of the Nb–Si–B ternary [28] calculated the Nb_3_B_2_ to be stable at 1600 °C and showed that it formed two three-phase equilibria Nb + T2 + Nb_3_B_2_ and T2 + Nb_3_B_2_ + NbB with neighbouring phases. Sun et al. [28] stated “there are no direct experimental evidences to support the existence of these two three-phase equilibria” and “further experimental results are needed to accurately define the stability region of Nb_3_B_2_”.

The Nb_3_B_2_ compound was reported by the same researchers [11] in the B-rich, arc melted and heat-treated (1500 °C/100 h) very small buttons (0.006 kg) of seven Nb–Ti–Si–B alloys, of which six were B-rich (B ≥ 10 at.%) and one B-lean (B = 1.5 at.%), all alloys had 16 at.%Si, four had Ti/Si < 1 (range 0.28 to 0.9, Ti = 4.5, 6, 12, 14.5 at.%) and three had Ti/Si > 1 (range 1.1 to 1.47, Ti = 18, 20, 23.5 at.%). The microstructures of the alloys were studied using EPMA and XRD. One of their alloys, namely Nb-23.5Ti-16Si-10B, was close to the actual composition of the alloy TT1 [12] and its microstructure was reported to consist of Nb_ss_, T2, Nb_3_Si and Nb_3_B_2_ compared with Nb_ss_, T2 and Nb_3_Si for TT1. It should also be noted that the Nb_3_B_2_ compound was not reported in the microstructure of the Nb-22Ti-16Si-3Al-5Cr-4Hf-10B (at.%) alloy [29]. According to the EPMA data of Sun et al. [11], in the seven Nb-Ti-Si-B alloys that they studied, the Nb_3_B_2_ had 39.7 < Si + B < 42.2 at.% (average 41 at.%), 0.5 < Si/B < 0.76 (average 0.64), 1.9 < Ti < 11.4 at.% (average 6.2 at.%) and 1.37 < (Nb + Ti)/(Si + B) < 1.52 (average 1.44). The T2 silicide had 33.8 < Si + B < 38.9 at.% (average 37.2 at.%), 1 < Si/B <8.3 (average 3.5), 6.5 < Ti < 18 at.% (average 14 at.%) and 1.6 < (Nb + Ti)/(Si + B) < 2 (average 1.7). Differences for the Si/B ratio and Ti content in Nb_3_B_2_ and D8_8_, and Ti content in T2, for the alloys studied by Sun et al. [11] and this work should be noted. In all four alloys of this work, the T2 was the primary phase. Considering the data for the alloy TT1 [12] and this work, there was two-phase Nb_ss_ and T2 equilibria in TT3, three-phase Nb_ss_, T2 and Nb_3_Si equilibria in TT1 and three-phase Nb_ss_, T2 and D8_8_ equilibria in TT8. Only the synergy of B with Al and Al + Cr in the alloys TT3 and TT4 was able to control the contamination of the microstructure by nitrogen (Table 4).

**Figure 4:**
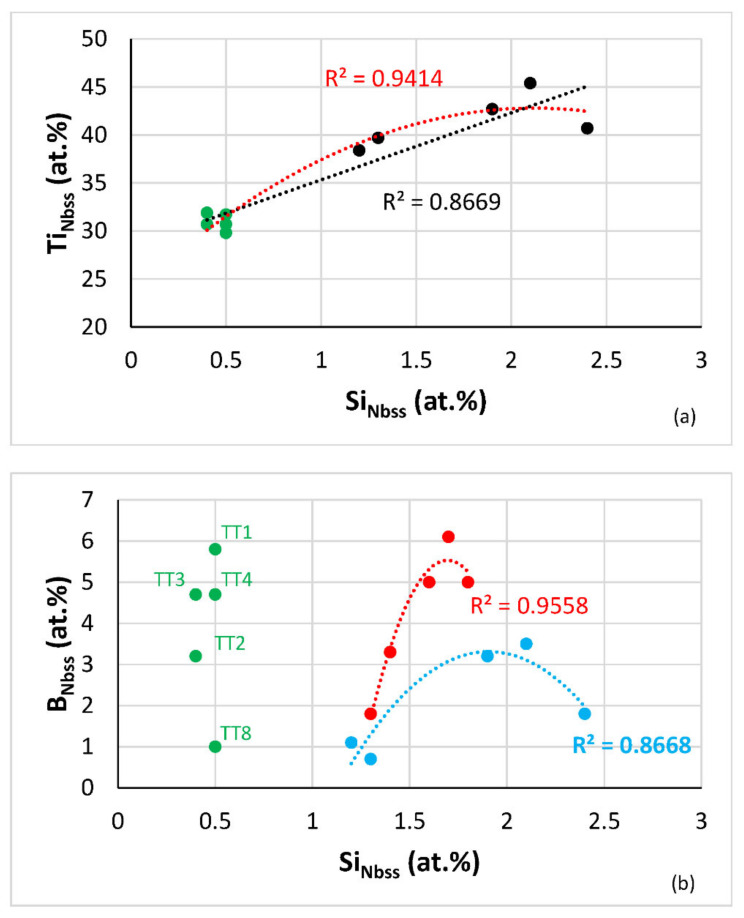

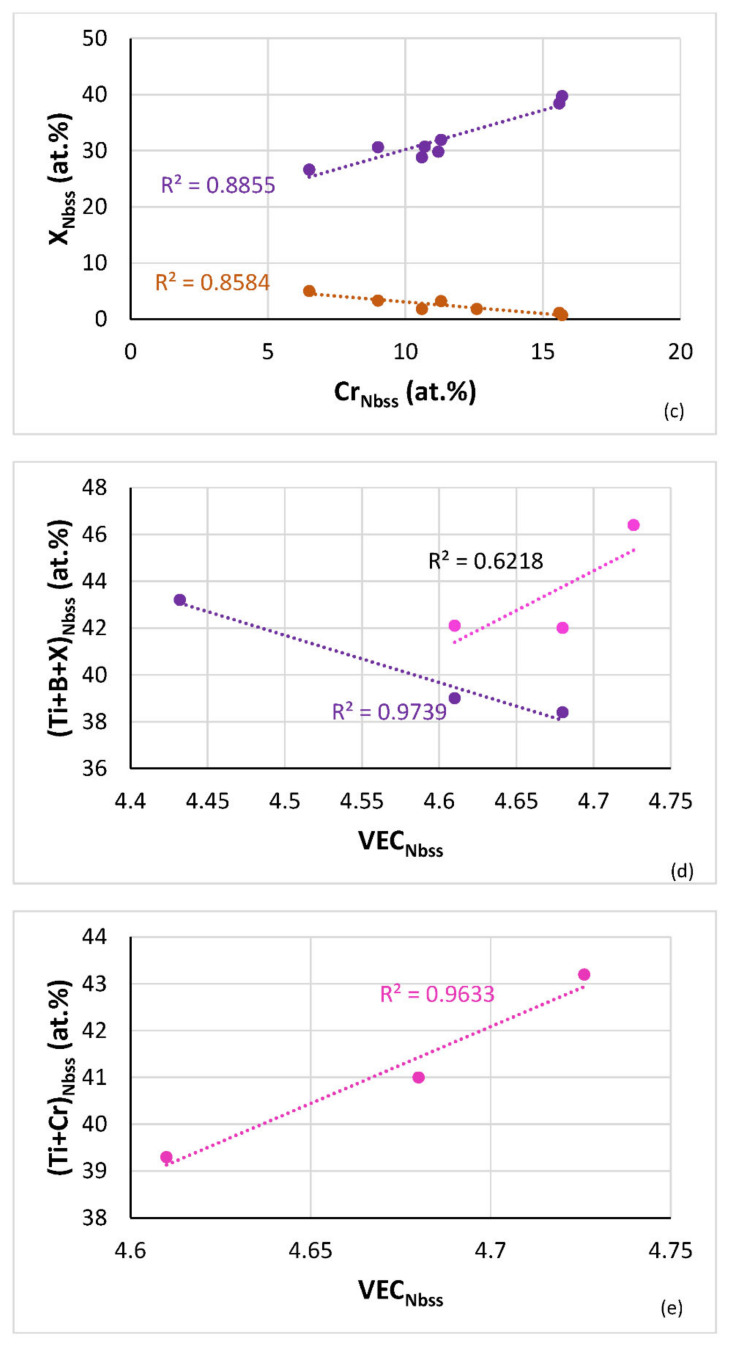

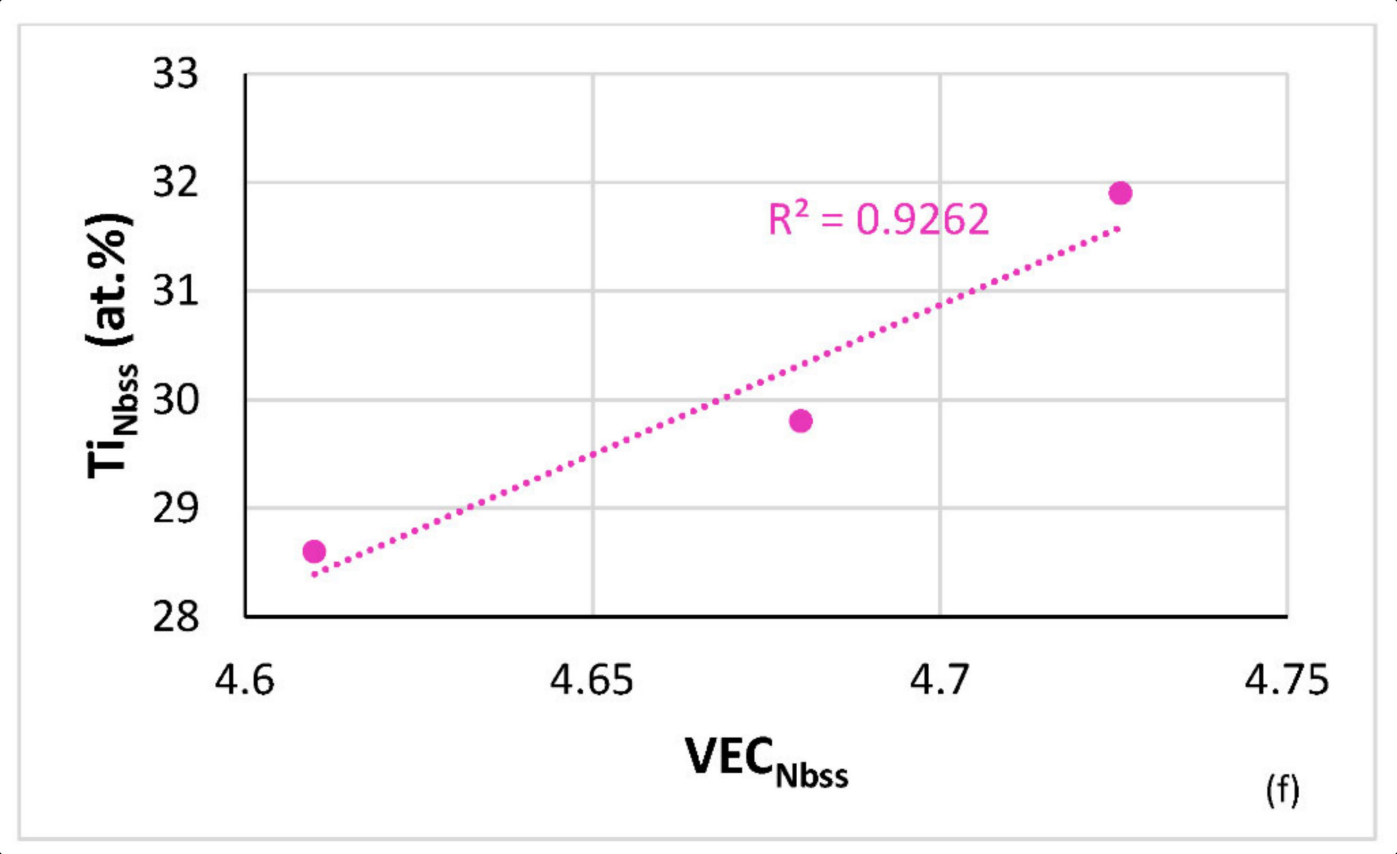
(**a**) Ti versus Si and (**b**) B versus Si content of the Nb_ss_ in the alloys TT1 [12], TT2, TT3, TT4 and TT8 where the data for HT alloys is shown in green. In (**a**) the R^2^ values are given for the linear or parabolic fit of data and in (**b**) the R^2^ values are for the parabolic fit of data, where blue filled circles correspond to Ti-rich Nb_ss_ and red to normal Nb_ss_ in AC alloy. (**c**) Element X versus Cr in Nb_ss_, data for X = B (brown) or Ti (purple) in the Nb_ss_ of the HT alloys TT2, TT4 and TT8. (**d**) Ti + B + X (X = Al, Cr) versus VEC of Nb_ss_ in the HT alloys TT2, TT3, TT4 and TT8, purple X = Al, alloys TT3, TT4, TT8, pink X = Cr, alloys TT2, TT4, TT8, (**e**) Ti + Cr versus VEC and (**f**) Ti versus VEC of Nb_ss_, HT alloys TT2, TT4, TT8.

The Si content in the Nb_ss_ increased with its Ti concentration (Figure 4a) and was essentially the same for all the HT alloys, about 0.5 at.%, the latter in agreement with other work on B-free RM(Nb)ICs e.g., [16,17]. Whereas the Si concentration was the same, the B content on the HT alloys varied depending on element(s) in synergy with B (Figure 4b). In the Ti-rich Nb_ss_ and “normal” Nb_ss_ the maximum B concentration respectively was about 6.1 and 3.2 at.%, which corresponded respectively to 1.7 and 1.9 at.%Si. The concentrations of B, Cr and Ti depended on each other, the Ti and B concentrations in the Nb_ss_ respectively increased and decreased with increasing Cr content in the Nb_ss_ (Figure 4c). As the Al + B + Ti content of the Nb_ss_ decreased the VEC of the solid solution increased, whereas the opposite was the case regarding B + Cr + Ti (Figure 4d). The VEC of the Nb_ss_ increases with Ti + Cr or Ti content in the alloys TT2, TT4 and TT8 (Figure 4e,f). The B or Mo content of the Nb_ss_ decreased with increasing Ti concentration in the Nb_ss_ (Table 5).

**Figure 5:**
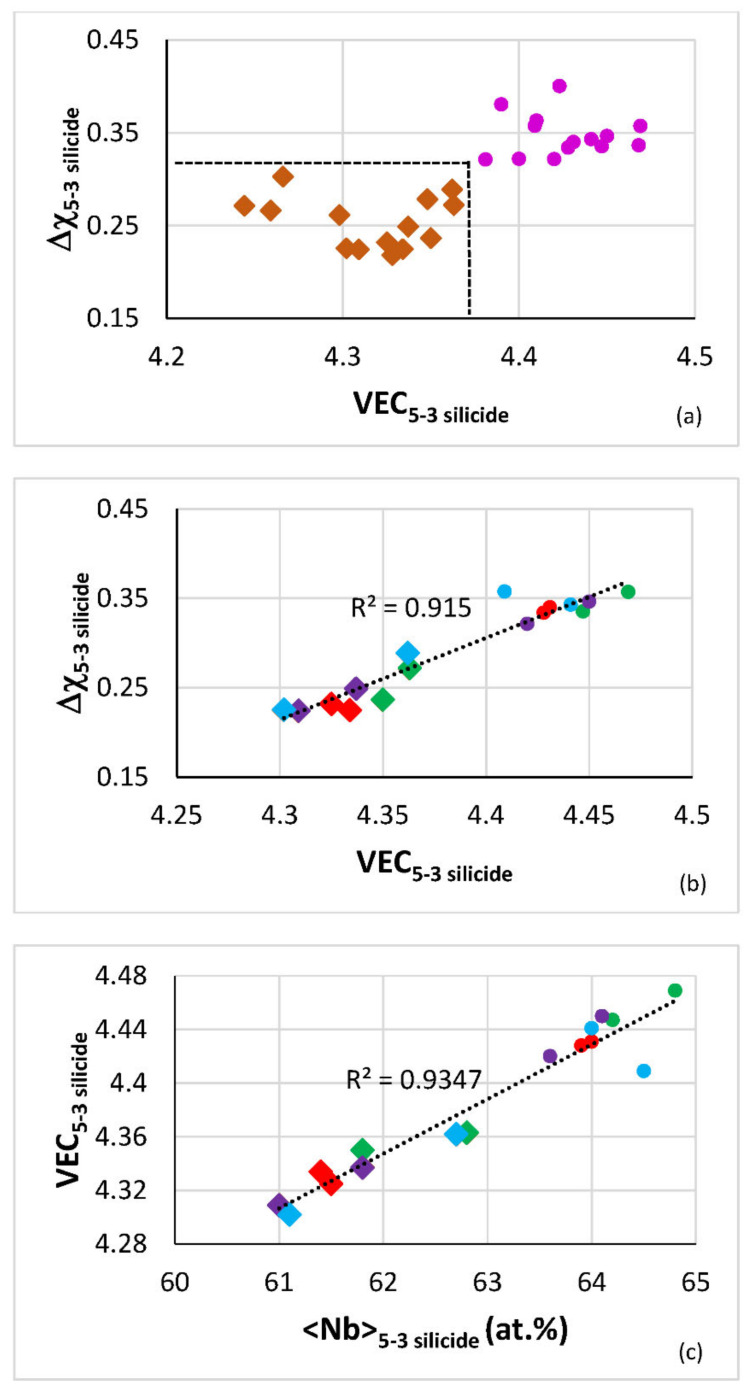

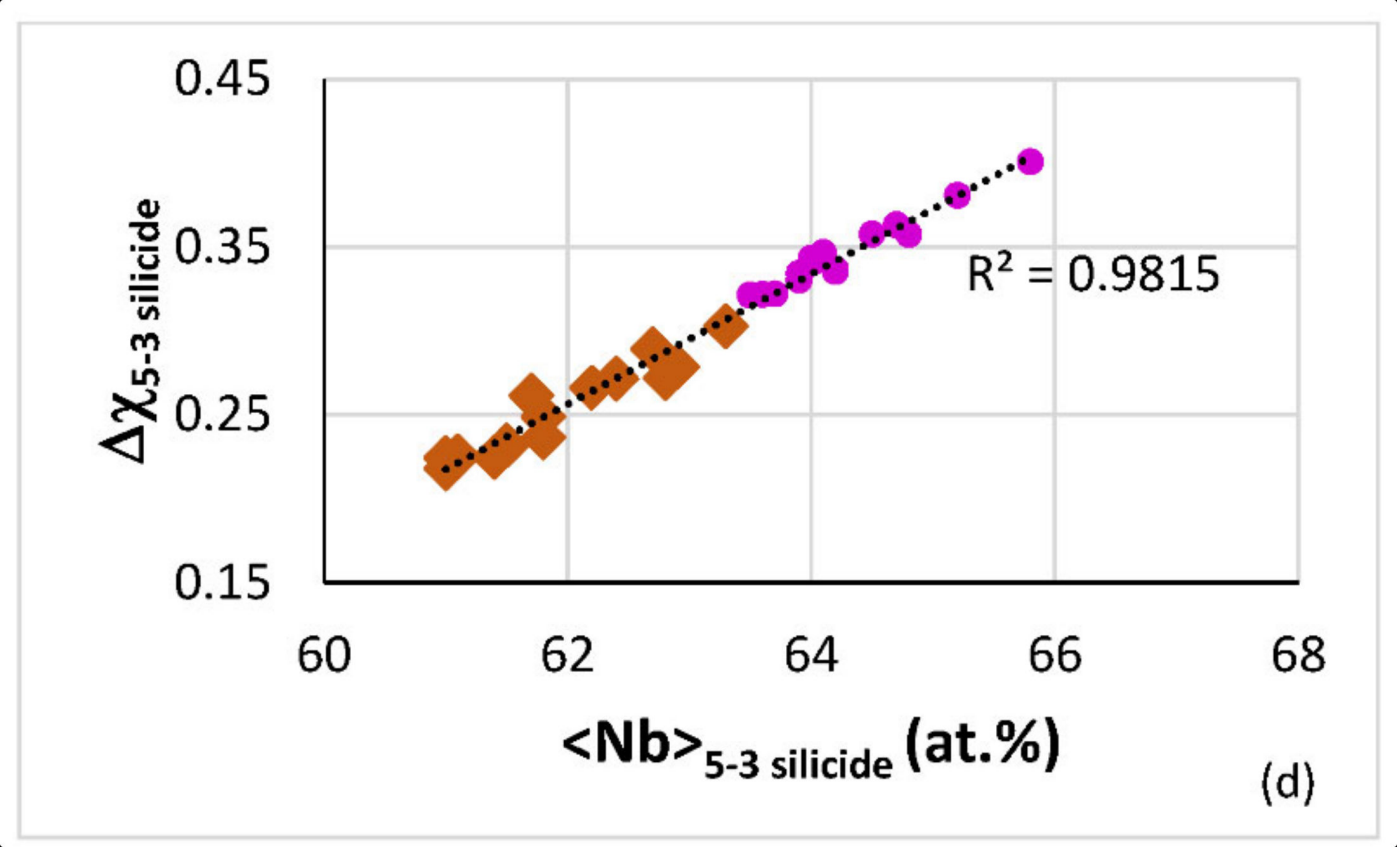
Maps of the 5-3 silicide in the AC and HT boron-containing alloys TT2, TT3, TT4 and TT8, i.e., of the T2 silicide, and the reference B-free alloys KZ4, KZ5, KZ7 and JG3, i.e., of Nb_5_Si_3_. (**a**) and (**b**) Δχ versus VEC, (**c**) VEC versus <Nb> and (**d**) Δχ versus <Nb>. Diamonds for B-containing alloys, i.e., data for T2, and pink filed circles for the B free alloys, i.e., data for Nb_5_Si_3_. (**a**) and (**d**) data for T2, Ti-rich T2, Nb_5_Si_3_ and Ti-rich Nb_5_Si_3_. (**b**) and (**c**) data for T2 and Nb_5_Si_3_ in the aforementioned alloys. In (**b**) and (**c**) green for TT2 and KZ4, red for TT3 and KZ7, purple for TT4 and KZ5, and blue for TT8 and JG3. <Nb> means Nb and other elements that substitute Nb in silicide. For all data in (**a**) R^2^ = 0.5173 for linear fit. The dashed lines in (**a**) delineate the “territory” occupied by the T2 silicide in the B containing alloys TT2, TT3, TT4 and TT8 (defined by Δχ and VEC approximately less than 0.303 and 4.36).

In the T2 silicide, the B content decreased and the Al and Cr concentrations increased as the Ti concentration increased ([6] and Table 5). Figure 5 shows maps of the T2 silicide in the AC and HT alloys TT2, TT3, TT4 and TT8 and of the Nb_5_Si_3_ silicide in the reference B-free alloys KZ4, KZ5, KZ7 and JG3. The T2 occupies a distinct, separate area in the Δχ versus VEC map (Figure 5a,b) in agreement with [2,6]. Both VEC and Δχ increase with increasing <Nb> and the linear fit of the data for T2 and Nb_5_Si_3_ is remarkably good (Figure 5c,d).

### 4.3. Hardness

**Figure 6:**
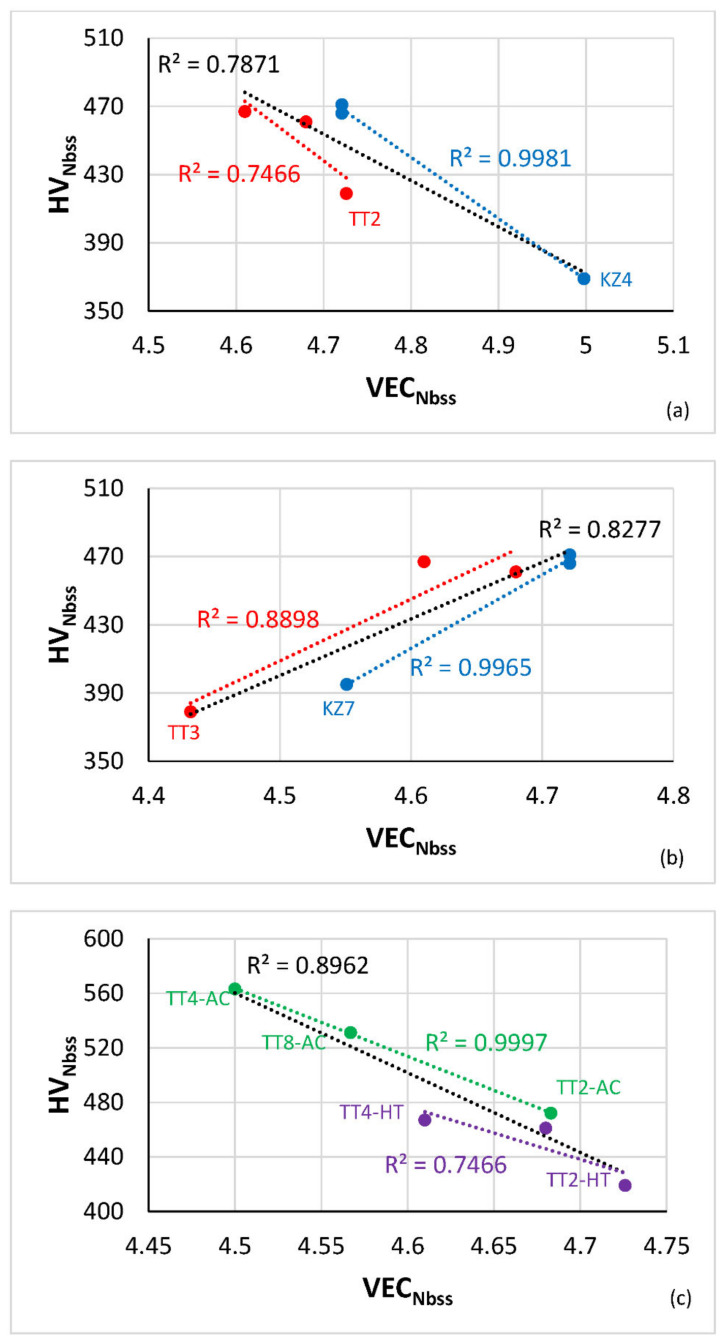
Vickers hardness (HV) of Nb_ss_ in HT alloys versus the parameter VEC of the solid solution. (**a**) alloys TT2, TT4, TT8 (red), KZ4, KZ5 and JG3 (blue), all data R^2^ = 0.7871 (**b**) alloys TT3, TT4, TT8 (red), KZ7, KZ5 and JG3 (blue), all data R^2^ = 0.8277. (**c**) Vickers hardness of Nb_ss_ in AC (green) and HT (purple) alloys TT2, TT4, TT8, all data R^2^ = 0.8962.

The hardness of the Nb_ss_ decreased or increased with the parameter VEC_Nbss_ when the alloys TT4 and TT8 were grouped respectively with the Cr containing alloy TT2 (Figure 6a) or the Al-containing alloy TT3 (Figure 6b). The same was the case for the reference alloys KZ5 and JG3 when grouped with the Cr or Al-containing alloys KZ4 and KZ7, respectively (Figure 6). The hardness of the Nb_ss_ in the AC and HT alloys TT2, TT4 and TT8 decreased with increasing VEC_Nbss_ (Figure 6c).

**Figure 7:**
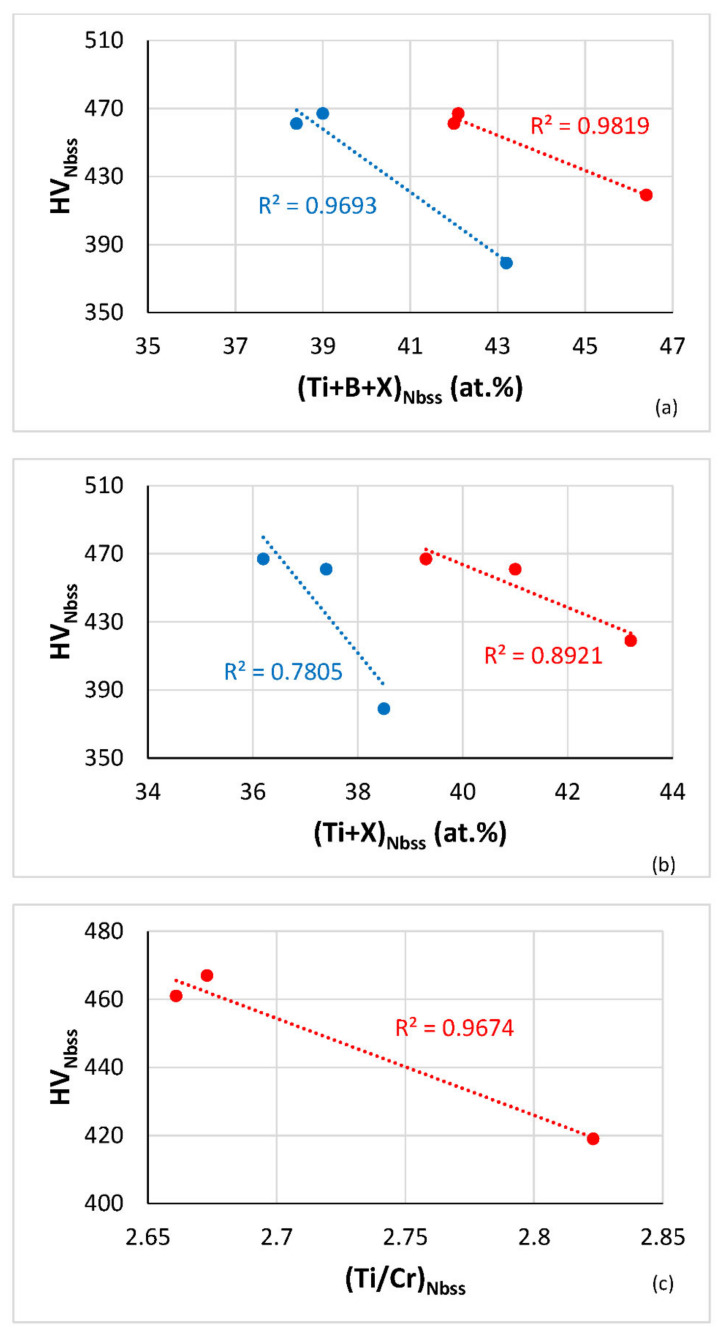

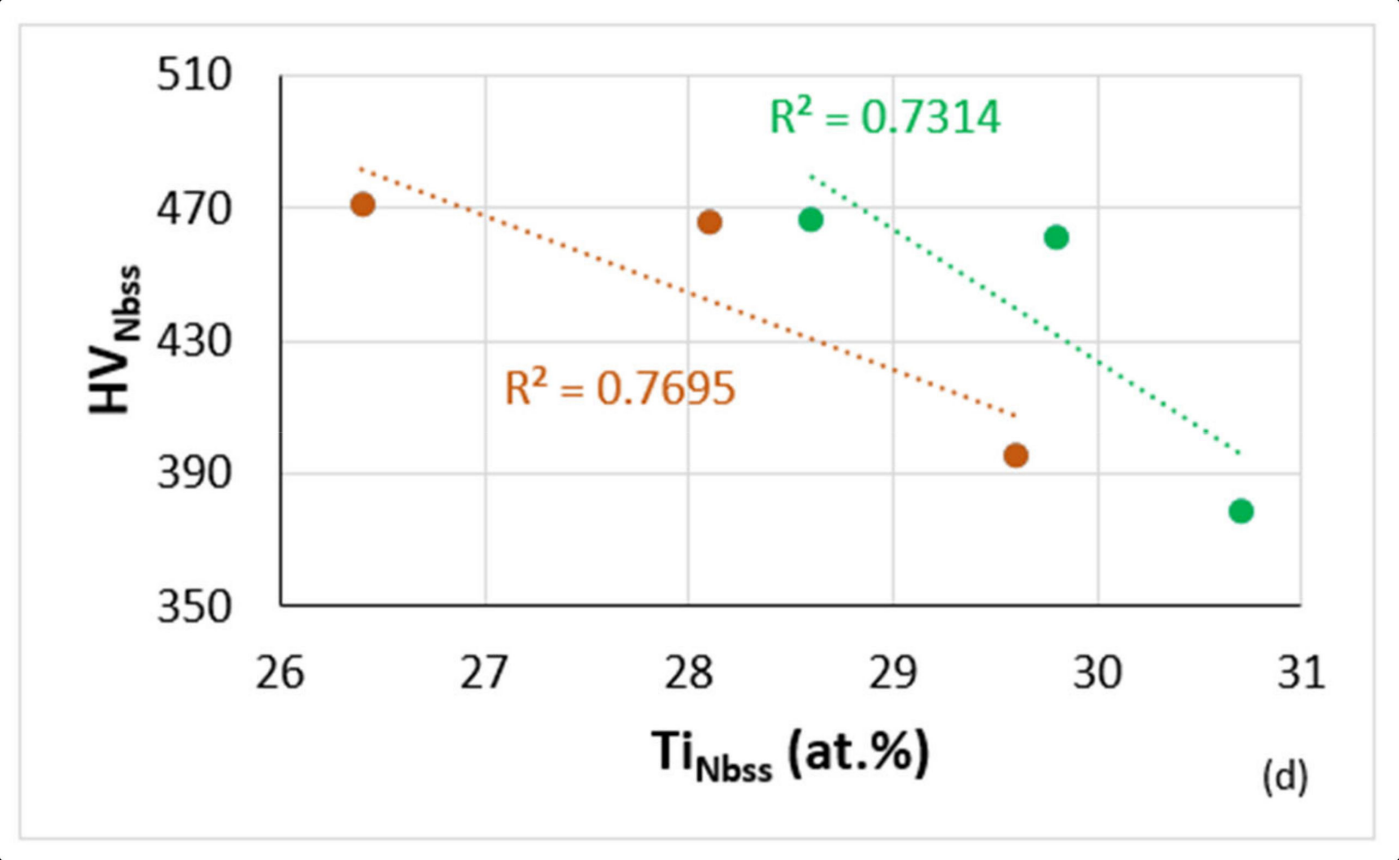
Vickers hardness (HV) of Nbss in the HT alloys TT2, TT3, TT4, TT8 versus (**a**) Ti + B + X, (**b**) Ti + X, (X = Al (blue), Cr (red)), (**c**) Ti/Cr for the alloys TT2, TT4, TT8, and (**d**) HV of Nbss in the HT alloys TT3, TT4, TT8 (green) and KZ7, KZ5 and JG3 (brown).

The dependence of the hardness of the Nb_ss_ on solutes in the alloys is shown in Figure 7. The hardness decreased with increasing Ti + B + X or Ti + X content (X = Al, Cr), and this was linked with changes of the parameter VEC_Nbss_ (Figure 4 and Figure 6), and also decreased with increasing Ti content in the Nb_ss_, the same trend as for the reference alloys (Figure 7d). Note the link between Ti in the solid solution and its VEC (Figure 4f) and the change of hardness with VEC (Figure 6a).

The VEC_T2_ or Δχ_T2_ increased with increasing <Nb> in T2 (Figure 5c,d) and the hardness of T2 increased with VEC_T2_ (in [2]). The synergy of Cr with B and Ti decreased slightly the hardness of T2 in TT2-AC, which was the highest amongst the AC alloys of this study, compared with TT1-AC, but the T2 in TT2-HT did not retain its hardness after heat treatment, which decreased by 13% compared with 6% in TT1-HT ([12] and Table 2) and was essentially the same as the hardness of T2 in TT3-HT and the second-lowest amongst all the HT alloys (Table 2). Best hardness retention of the T2 and highest hardness after heat treatment was observed in the case of the alloy TT4. Compared with the alloys ZF4 [30], ZF5 [31] and ZF6 [32], which are similar respectively to TT2, TT3 and TT4 with the addition of Ge instead of B, the T2 had significantly lower hardness than Nb_5_Si_3_ in these three alloys. Compared with TT1, the T2 hardness in TT4-HT was not significantly different than that in TT1-HT [12], which was also lower than the Nb_5_Si_3_ in ZF3 [33]. It should be noted that the relationship between the hardness of T2 and VEC_T2_ is opposite that of Nb_5_Si_3_ and VEC_Nb5Si3_ (in [2]).

**Figure 8:**
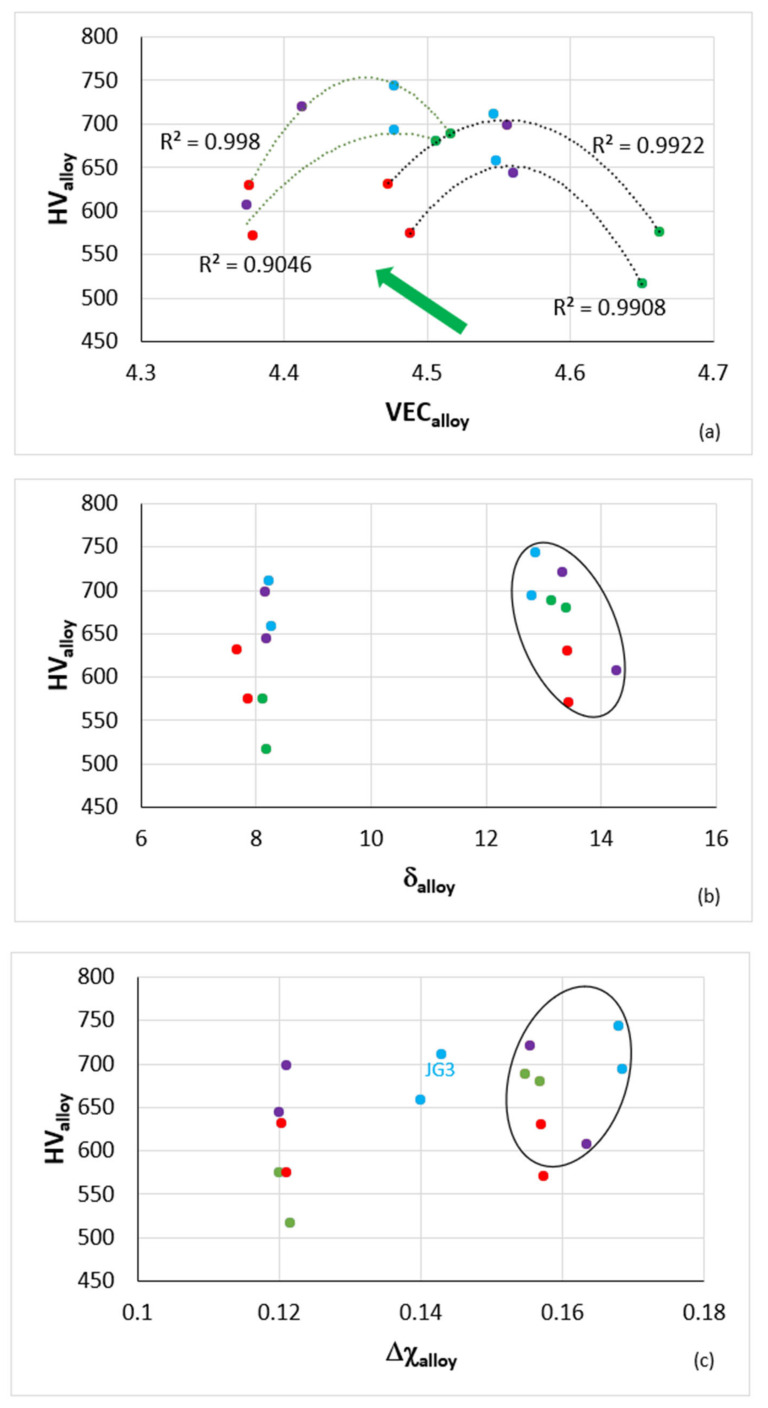

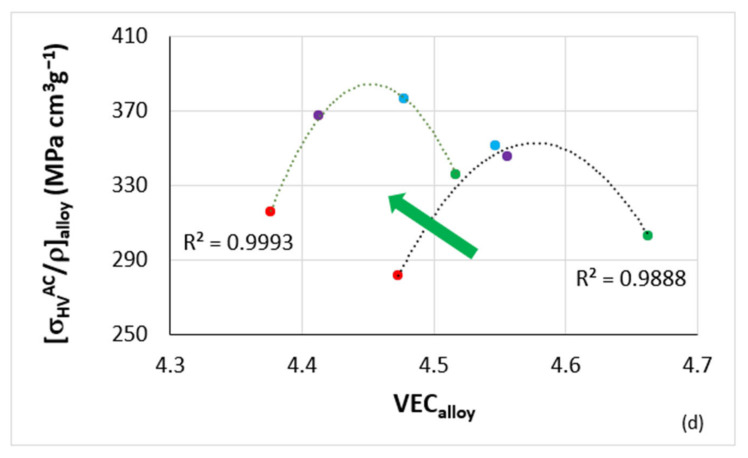
(**a**) to (**c**) Vickers hardness (HV) and (**d**) room temperature specific strength calculated from hardness (σ_HV_/ρ) versus the parameters (**a**), (**d**) VEC, (**b**) δ and (**c**) Δχ of the TT2, TT3, TT4, TT8, KZ4, KZ5, KZ7 and JG3 alloys. In (**a**) to (**c**) the data is for the AC and HT conditions and in (**d**) the data is for the AC condition. Filled circles green TT2 and KZ4, red TT3 and KZ7, purple TT4 and KZ5, and blue TT8 and JG3. In (**b**) and (**c**) ellipses enclose the B containing alloys. In (**a**) and (**d**) the R^2^ values are for the parabolic fit of data. In (**a**) data for the alloys TT2, TT3, TT4 and TT8 with R^2^ = 0.998 and R^2^ = 0.9046 for the AC and HT conditions, respectively and with R^2^ = 0.9922 and R^2^ = 0.9908 respectively for the AC and HT alloys KZ4, KZ5, KZ7 and JG3. In (**d**) R^2^ = 0.9993 and R^2^ = 0.9888 respective for the AC alloys TT2, TT3, TT4, TT8 and KZ4, KZ5, KZ7, JG3. In (**a**) and (**d**) green arrows indicate the effect of B addition.

Alloy hardness versus the parameters VEC_alloy_, δ_alloy_ and Δχ_alloy_ is shown in Figure 8a–c. Room temperature specific strength calculated from hardness (σ_HV_/ρ)_alloy_ is shown in Figure 8d. The addition of B to the reference alloys caused a decrease in VEC_alloy_ and an increase in HV_alloy_ and (σ_HV_/ρ)_alloy_. Notice that the B containing alloys occupy specific distinct areas in the HV_alloy_ versus δ_alloy_ or Δχ_alloy_ maps.

Furthermore, notice the location of the JG3 alloy in the HV_alloy_ versus Δχ_alloy_ map. The maximum specific strength of 385 MPa cm^3^g^−1^ for VEC_alloy_ = 4.45 from the parabola for B containing alloys in Figure 8d is significantly higher than those reported for RCCAs to date [1,2], and only slightly lower than that of the RM(Nb)ICs-RCCAs JZ4 and JZ5 [34].

**Figure 9:**
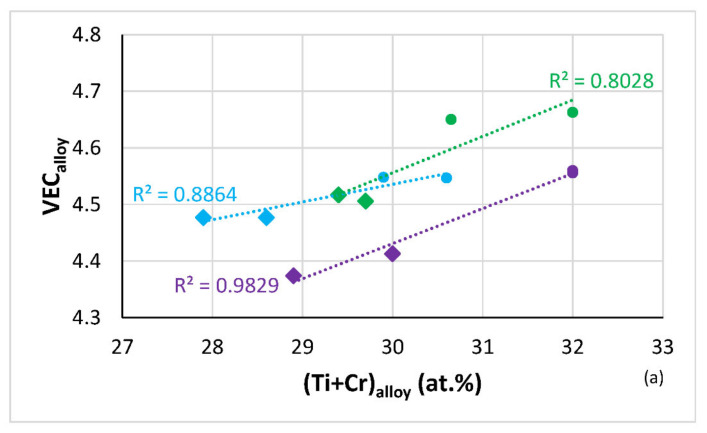

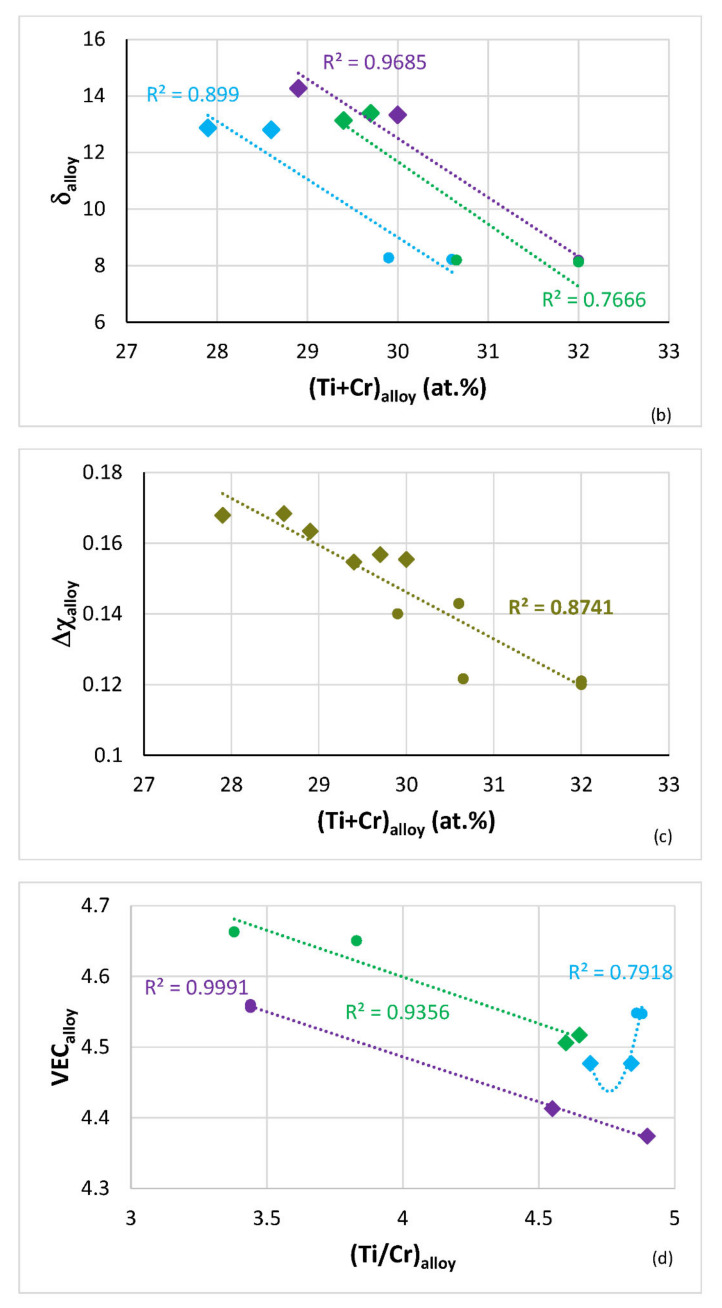

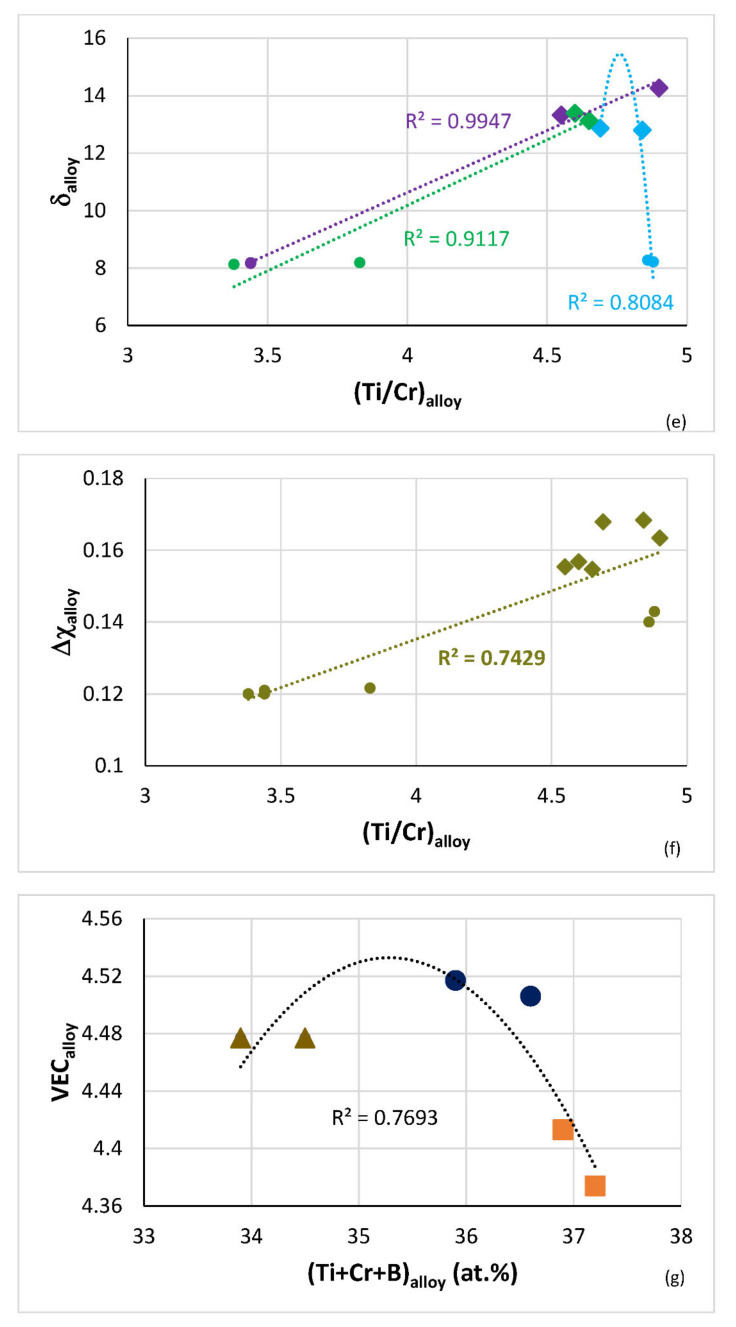

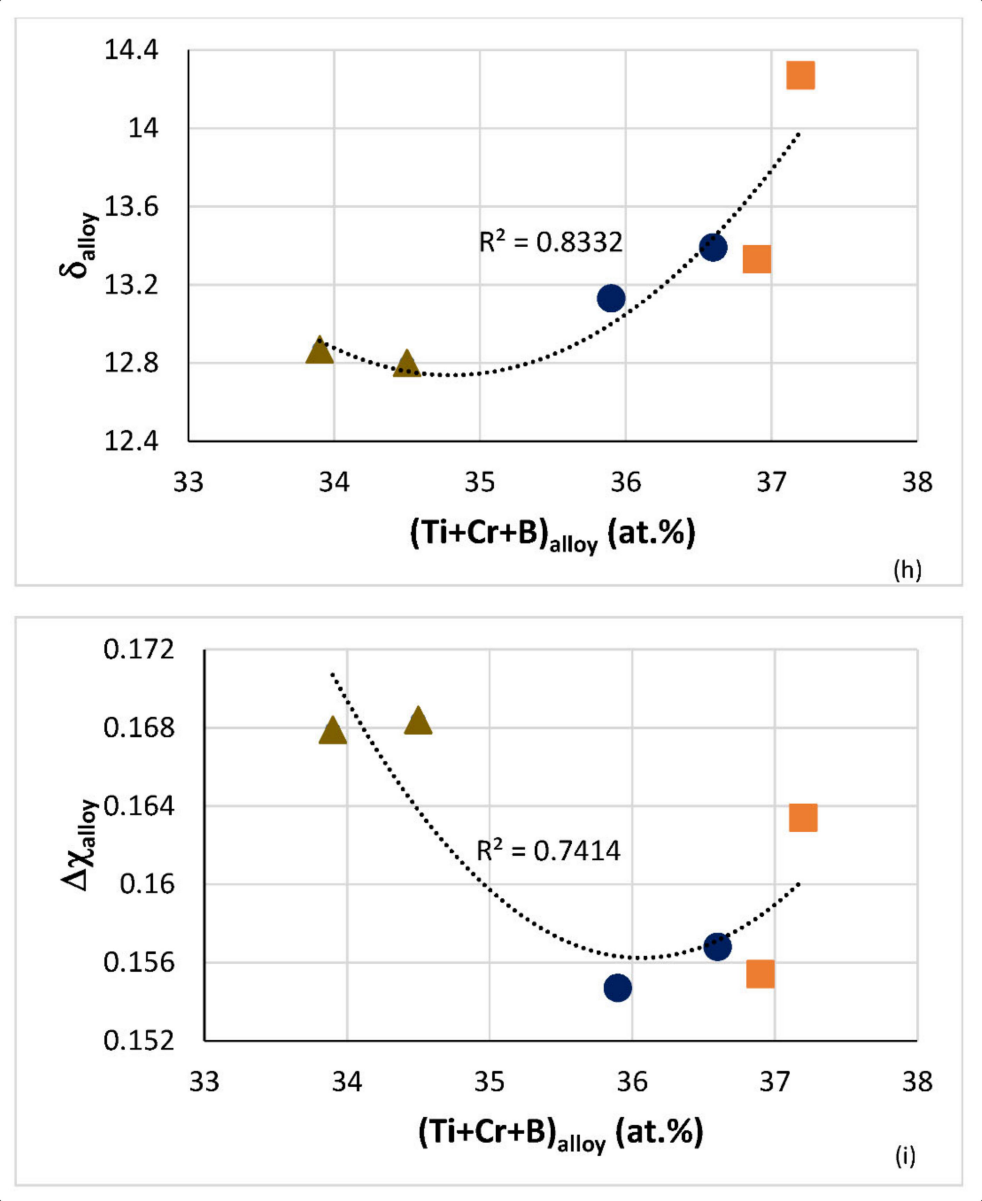
Maps of the parameters VEC_alloy_, δ_alloy_ and Δχ_alloy_ with sums or ratios of Ti, Cr or B in AC and HT alloys TT2, TT4, TT8 and “equivalent” B free alloys KZ4, KZ5 and JG3. R^2^ values for linear or parabolic fit of data. (**a**) VEC_alloy_ versus (Ti + Cr)_alloy_, (**b**) δ_alloy_ versus (Ti + Cr)_alloy_, (**c**) Δχ_alloy_ versus (Ti + Cr)_alloy_, (**d**) VEC versus Ti/Cr, (**e**) δ versus Ti/Cr, (**f**) Δχ versus Ti/Cr, (**g**) VEC versus Ti + Cr + B, (**h**) δ versus Ti + Cr + B and ((**i**) Δχ versus Ti + Cr + B. (**a**) to (**f**) data for the alloys TT2, KZ4, TT4, KZ5, TT8 and JG3, (**g**) to (**i**) data for the alloys TT2, TT4, TT8. (**a**,**b**,**d**,**e**) colours as follows: green for TT2 and KZ4, purple for TT4 and KZ5, and blue for TT8 and JG3. (**g**) to (**i**) triangles for TT8, squares for TT4, and circles for TT2. (**c**) and (**f**) green colour for all alloys. In (**a**) to (**f**) diamonds for B containing alloys.

The parameters VEC_alloy_, δ_alloy_ and Δχ_alloy_ link with specific solutes and thus allow us (i) to link the hardness and specific strength of alloys with solute additions and; (ii) to use these relationships for the design of alloys using the alloy design methodology NICE [1,2,3]. We demonstrate such relationships for the alloys TT2, TT4 and TT8 and the solutes B, Cr and Ti in Figure 9. 

**Figure A1:**
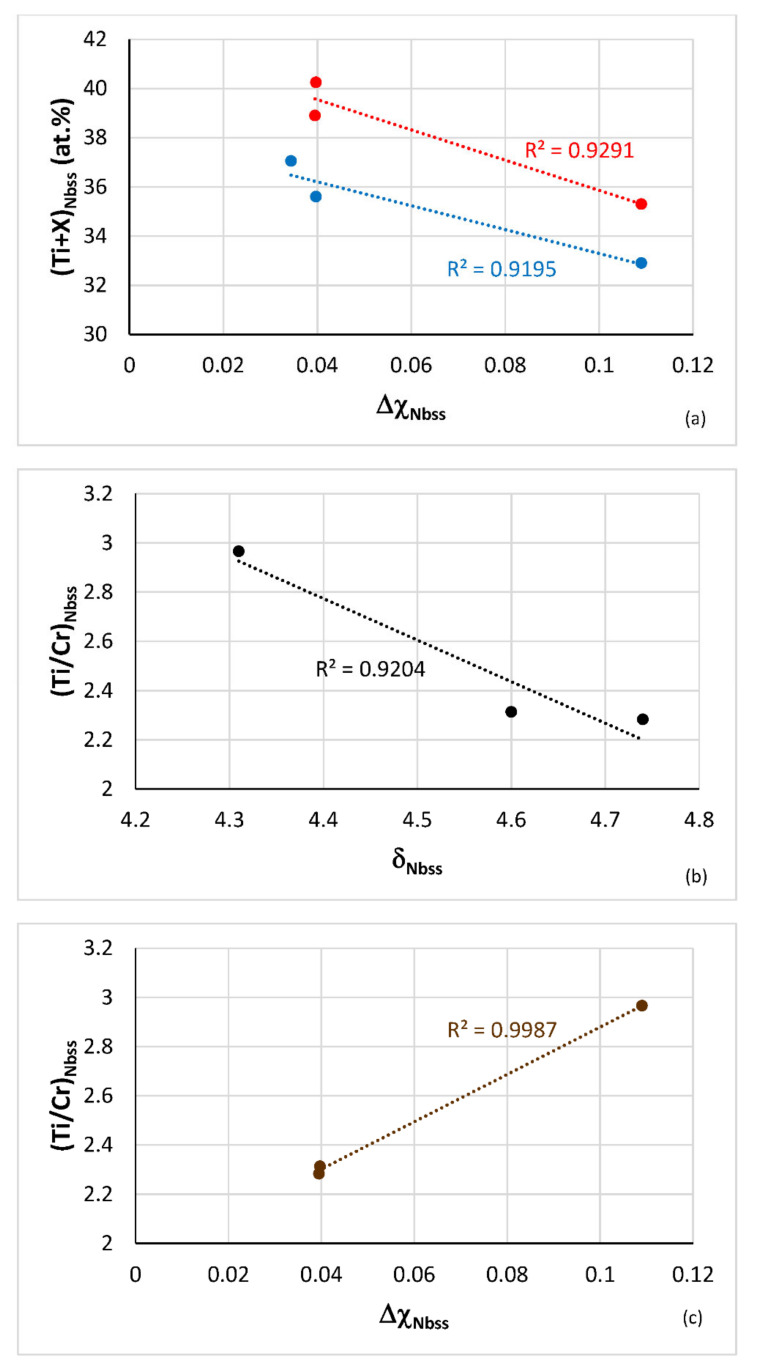
Data for the Nb_ss_ in HT boron free alloys KZ4, KZ5, KZ7 and JG3. (**a**)Ti + X (X = Al (blue), Cr (red)) versus the parameter Δχ for the alloys KZ4, KZ5, KZ7 and JG3, (**b**) and (**c**) Ti/Cr versus (**b**) δ for the alloys KZ4, KZ5 and JG3 and (**c**) Δχ for the alloys KZ7, KZ5 and JG3.

The parameter VEC_alloy_ increased with (Ti + Cr)_alloy_ (Figure 9a) whereas the parameters δ_alloy_ and Δχ_alloy_ decreased (Figure 9b,c). The shift towards higher HV_alloy_ with decreased VEC_alloy_ for the B-containing alloys (Figure 8a) was thus attributed to the lower (Ti + Cr) content in the alloys TT2, TT4 and TT8. The shift of the latter in separate distinct areas in the maps in Figure 8b,c was attributed to the increase of δ_alloy_ and Δχ_alloy_ with the decrease of (Ti + Cr)_alloy_ (Figure 9b,c). The parameter VEC_alloy_ decreased and the δ_alloy_ and Δχ_alloy_ increased with increasing (Ti/Cr)_alloy_ (Figure 9d,e,f). The alloys were separated in the VEC_alloy_ or δ_alloy_ versus (Ti/Cr)_alloy_ maps, as was the case in the VEC_alloy_ or δ_alloy_ versus (Ti + Cr)_alloy_ maps, but in the former two maps the case for Mo-containing alloys JG3 and TT8 was different, with VEC_alloy_ minimum at about 4.45 and δ_alloy_ maximum at about 15 for (Ti/Cr)_alloy_ = 4.75. Note that VEC_alloy_ ≈ 4.45 corresponds to maximum HV_alloy_ and specific strength in Figure 8a,d. Similarly to the Δχ_alloy_ versus (Ti + Cr)_alloy_ map, the alloys were not separated in the Δχ_alloy_ versus (Ti/Cr)_alloy_ map (Figure 9c,f). Thus, even though there are relationships of the parameters VEC_alloy_, δ_alloy_ and Δχ_alloy_ with (Ti + Cr)_alloy_, or (Ti/Cr)_alloy_, and the parameters VEC_alloy_, δ_alloy_ and Δχ_alloy_ are used to design new alloys using NICE [1,2,3], only the parameter VEC_alloy_ links hardness or specific strength with specific solutes in NICE, (Ti + Cr)_alloy_ and (Ti/Cr)_alloy_ in the case demonstrated above. Furthermore, the parameters VEC_alloy_, δ_alloy_ and Δχ_alloy_ also link with the (Ti + Cr + B) content of the alloys TT2, TT4 and TT8, as shown in the maps in Figure 9g,h,i. VEC_alloy_ is maximum for (Ti + Cr + B)_alloy_ = 35.5 at.%. The HV_alloy_ versus (Ti + Cr + B)_alloy_ map (not shown) shows minimum (685 HV_alloy_) and maximum (740 HV_alloy_) hardness, respectively, for AC and HT alloys for Ti + Cr + B = 35.5 at.% whereas the HV_alloy_ versus (Ti + Al + B)_alloy_ map (not shown) shows minima of 615 HV_alloy_ and 560 HV_alloy_ with (Ti + Al + B)_alloy_ equal to 34.5 and 35.5 at.%, respectively, for the AC and HT alloys TT3, TT4 and TT8. It should be noted that also the hardness of the solid solution (HV_Nbss_) links with VEC_Nbss_ (Figure 6) and with solutes, namely Ti + X, Ti + B + X (X = Al, Cr), and Ti/Cr (Figure 7) and that solutes in the Nb_ss_ link with its parameters (see Figure 4 for relationships with VEC_Nbss_ for B containing alloys and Figure A1 in the Appendix A for relationships in the reference alloys). Such relationships allow the design methodology NICE to optimise alloy composition for the balance of strength and oxidation properties, see [1,2,3].

**Figure 10:**
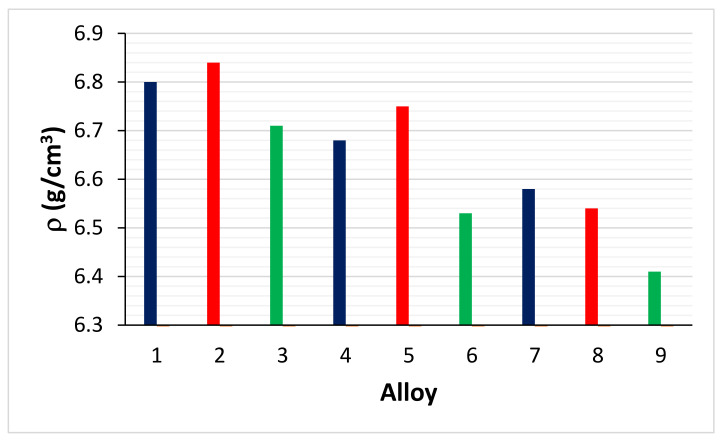
Densities of as cast alloys KZ4, KZ7, KZ5 (dark blue, 1, 4, 7), ZF4, ZF5, ZF6 (red, 2, 5, 8) and TT2, TT3, TT4 (green, 3, 6, 9). See Appendix A for nominal compositions of alloys.

Figure 10 compares the density of the base alloys KZ4, KZ7 and KZ5 with that of the equivalent alloys with Ge addition, namely ZF4, ZF5 and ZF6 [30,31,32], and with B addition.

**Figure 11:**
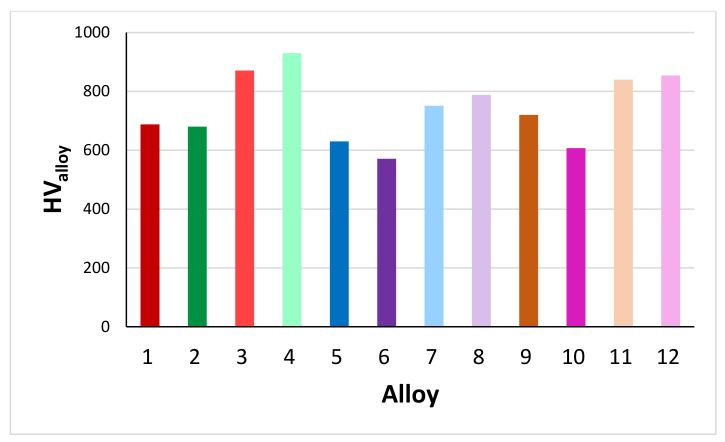
Room temperature Vickers hardness of AC and HT alloys. Odd numbers AC alloys, even numbers HT alloys. TT2 (1, 2), ZF4 (3, 4), TT3 (5, 6), ZF5 (7, 8), TT4 (9, 10), ZF6 (11, 12). See Appendix A for nominal compositions of alloys.

It is clear that the B addition had the most significant effect on reducing alloy density. Figure 11 compares the room temperature hardness of AC and HT alloys ZF4, ZF5, ZF6 [30,31,32] and TT2, TT3 and TT4. The addition of Ge to the reference alloys increased the alloy hardness compared with the addition of B. This was attributed (i) to the increase of the hardness of the Nb_5_Si_3_ when alloyed with Ge; and (ii) to the lower vol.% of Nb_ss_ in the Ge containing alloys [6,30,31,32] compared with the alloys TT2, TT3 and TT4.

### 4.4. Oxidation

The data for the alloys TT1, TT2, TT3, TT4 and TT8 can help us to infer what the effect of B addition to the reference alloys was on isothermal oxidation at 800 and 1200 °C and to compare the effect of B addition with that of Ge or Sn to the same reference alloys. Amongst the reference alloys KZ4, KZ5, KZ7 and JG3, the alloy JG3-AC had the best oxidation behaviour at both temperatures, did not pest at 800 °C, and followed parabolic oxidation at 800 °C and liner oxidation at 1200 °C (the JG3-HT followed linear oxidation at both temperatures) [35]. The rate constant of the alloys TT1, TT2 and TT3 at 800 °C (Table 6) was one order of magnitude higher than that of JG3-HT, and of the alloys TT4 and TT8 similar or one order of magnitude lower, respectively. The alloys KZ4, KZ5 and KZ7 formed Maltese crosses at 800 °C [36], like the MASC alloy, and their scales spalled off at 1200 °C, as did the scale of JG3.

At 800 °C, the mass change of TT1 was significantly lower than that of KZ3, which gained more than about 200 mg/cm^2^ after just 10 h. The synergy of B and Cr decreased the mass change compared with KZ4 (about 60 mg/cm^2^ after 100 h). The Al addition in TT3 also improved the oxidation behaviour compared with TT1 (Table 6). Compared with KZ7, the synergy of Al with B was only marginally beneficial for the first 20 h, after this time the mass change increased. The mass of the alloy TT4 did not change for the first 10 h and after this time it was lower than that of KZ5, which gained about 32.5 mg/cm^2^. The Mo addition in TT8 significantly improved the oxidation behaviour compared with JG3, which gained about 3.5 mg/cm^2^.

**Figure 12:**
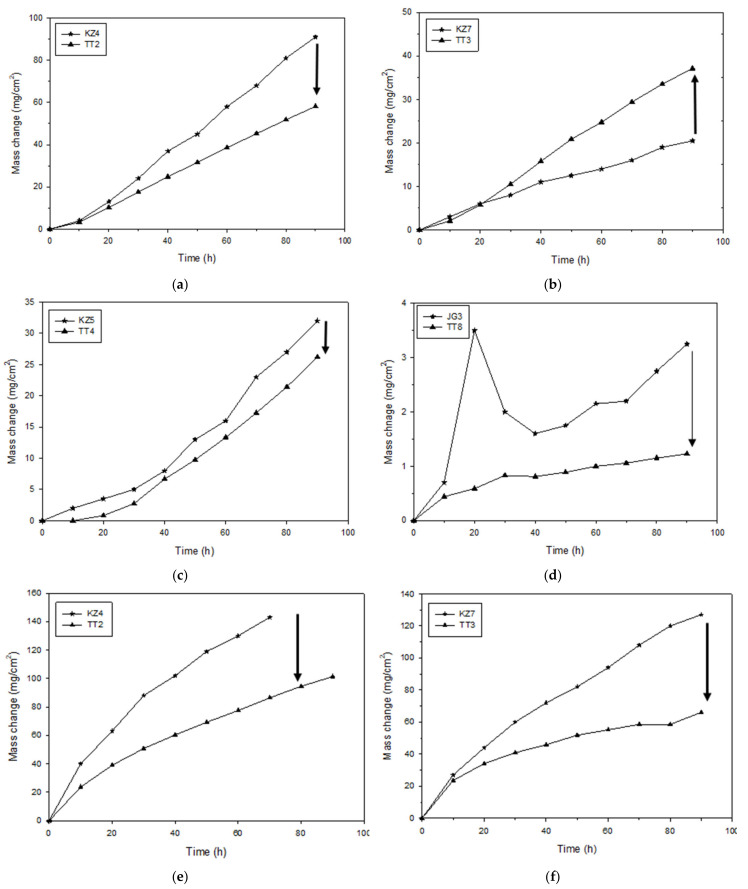

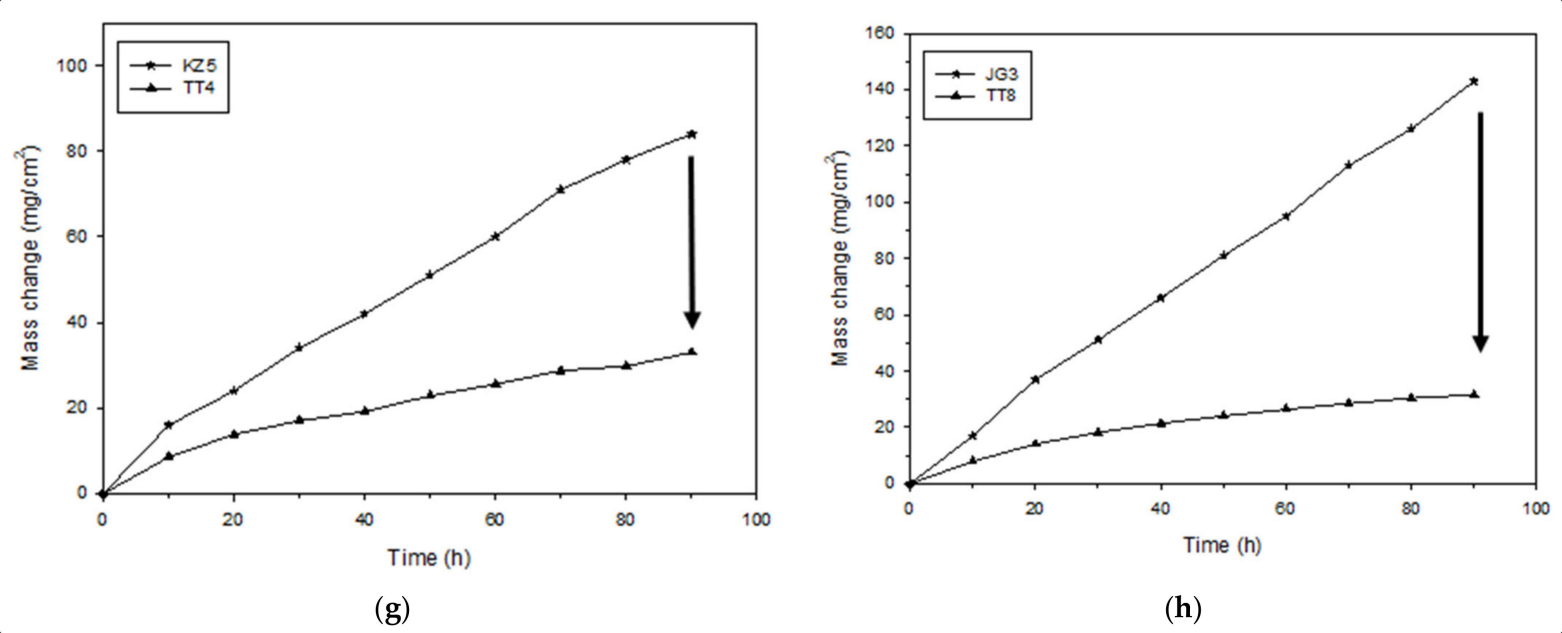
Comparison of mass changes of B-containing alloys TT2, TT3, TT4 and TT8 with reference alloys KZ4, KZ5, KZ7 and JG3. For isothermal oxidation at 800 and 1200 °C. Arrows indicate direction of mass change at 800 °C (**a**–**d**) and 1200 °C (**e**–**h**).

At 1200 °C, the synergy of Al and Cr in TT4 had a strong effect on oxidation behaviour (mass change after 90 h: TT1 = 80.3, TT2 = 105.6, TT3 = 66.1 and TT4 = 34.0 mg/cm^2^). The addition of Mo in the TT8 alloy resulted in a marginal improvement of the oxidation behaviour of TT8 compared with TT4. Figure 12 compares the mass changes of the alloys of this study with their reference alloys after 90 h at 800 or 1200 °C. In each part of this figure, the effect of B addition is indicated by an arrow. With the exception of TT3, whose mass increased more than KZ7 at 800 °C (Figure 12b), the addition of B resulted to lower mass changes at both temperatures, particularly in the case of the alloy TT8.

**Figure 13:**
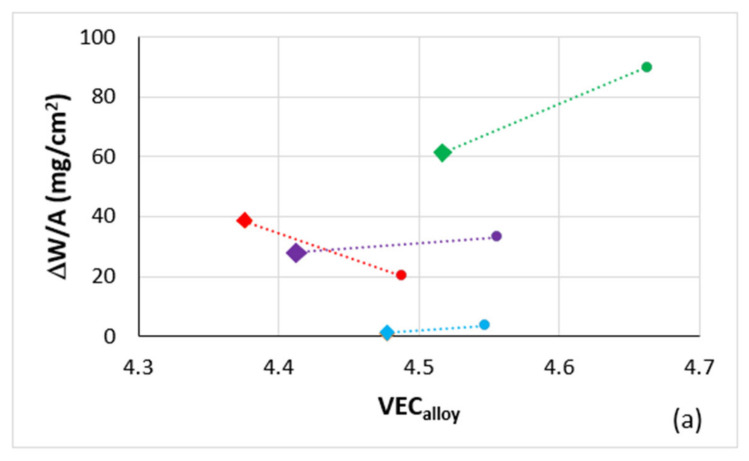

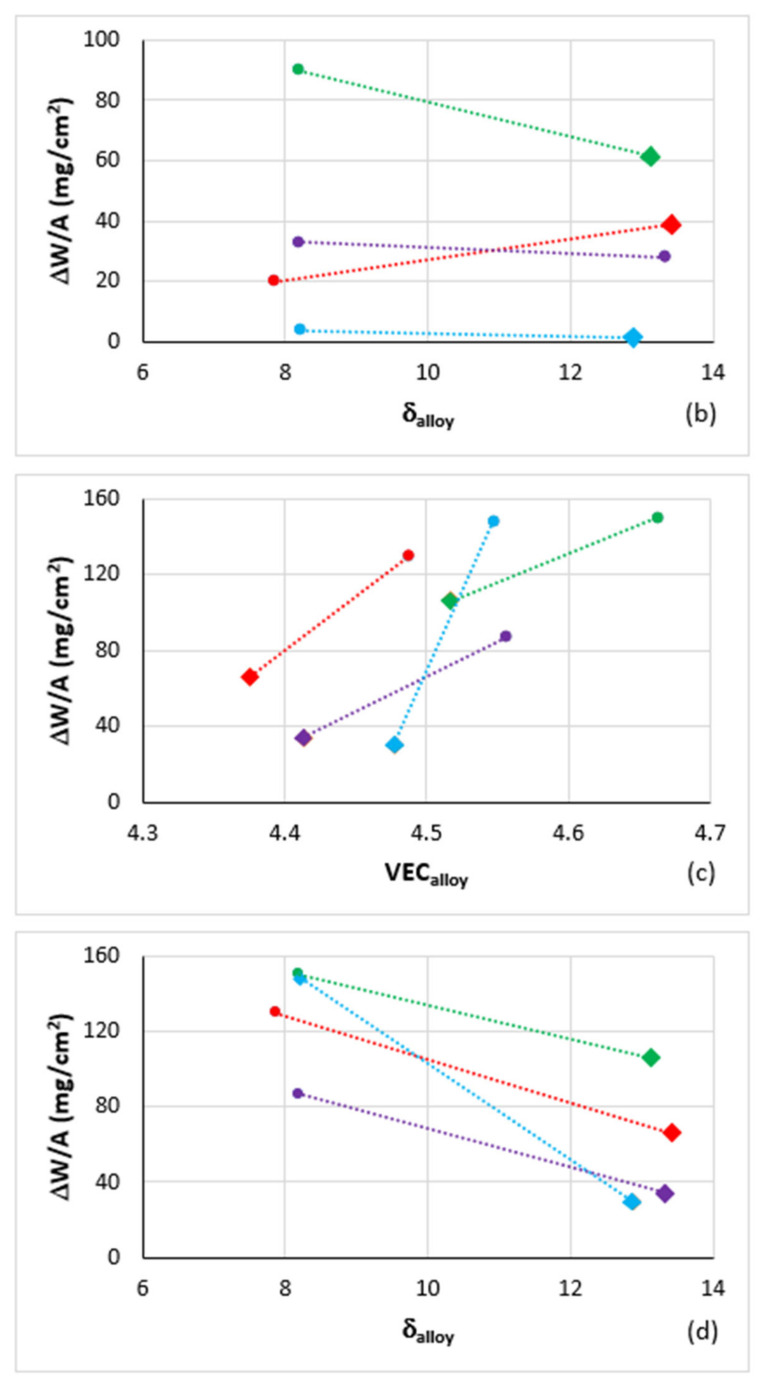
Mass change (ΔW/A) of the alloys TT2, TT3, TT4, TT8 and their basis/reference alloys KZ4, KZ5, KZ7 and JG3 versus the parameters (**a**,**c**) VEC and (**b**,**d**) δ for isothermal oxidation (**a**,**b**) at 800 °C and (**c**,**d**) at 1200 °C. Colours as follows: green for TT2 and KZ4, red for TT3 and KZ7, purple for TT4 and KZ5, and blue for TT8 and JG3. Diamonds for B containing alloys.

Plots of the mass changes of the B-containing alloys and their reference alloys versus the alloy parameters VEC and δ are shown in Figure 13. According to the alloy design methodology NICE, for improved oxidation behaviour, the alloy design should aim to lower VEC_alloy_ and increase δ_alloy_ [1,2,3]. Figure 13 shows that this was the case for all alloys at 1200 °C, and also at 800 °C where the alloy TT3 was the exception as its mass change was higher than that of KZ7 (Figure 12b and Figure 13a,b).

Compared with the Ge containing alloys ZF4, ZF5 and ZF6 [37] and the Sn containing alloys ZX4, ZX6 and ZX8 [22] (for nominal compositions see Table A1 in the Appendix A), the oxidation of the B-containing alloys TT2, TT3 and TT4 was inferior at 800 °C. Indeed, the above ZF and ZX series alloys did not pest, their scales did not spall off, and followed parabolic oxidation kinetics [37], or parabolic with/without linear kinetics [22]. At 1200 °C the ZF and ZX series alloys followed either parabolic plus linear kinetics [37] or parabolic or linear kinetics [22] and their scales spalled off, the same as the KZ series reference alloys. Only the alloy TT8 did not pest at 800 °C and its scale did not spall off at 800 and 1200 °C.

At 800 °C, the scales that formed on the alloys TT1 and TT2 consisted of SiO_2_, TiO_2_, Nb_2_O_5_, 3Nb_2_O_5_.TiO_2_, and 5Nb_2_O_5_.2TiO2, which are typical oxides in the scales of RM(Nb)ICs [3,35,36], plus B_2_O_3_ and B_2_SiO_5_ in TT1 plus CrNbO_4_ in TT2 (Figure S2a in Supplemental data). The scales that formed on TT3 and TT4 consisted of the same oxides as TT1, plus AlNbO_4_ in TT3 and CrNbO_4_ and AlNbO_4_ in TT4 (data not shown). At 1200 °C, the scale on TT1 consisted of SiO_2_, TiO_2_, Nb_2_O_5_ and 3Nb_2_O_5_.TiO_2,_ 5Nb_2_O_5_.2TiO_2_, plus B_2_O_3_ and B_2_SiO_5_ whereas the scale on TT2 consisted of the same oxides plus NbO, and CrNbO_4_ (Figure S2b in Supplementary Materials). The scale of TT3 consisted of the same oxides as TT1 plus AlNbO_4_ (data not shown). The presence of CrNbO_4_ and AlNbO_4_ in the scales of the alloys TT2, TT3 or TT4 is in agreement with the data for the B free alloys KZ4, KZ7 and KZ5 [3,36] and the Ge containing alloys ZF4, ZF5 and ZF6, where also SiO_2_, Nb_2_O_5_, TiNb_2_O_7_ (Nb_2_O_5_.TiO_2_) and GeO_2_ were observed [37]. 

Comparison of the oxidation of the reference alloys KZ4, KZ7, KZ5 [36] with the alloys with B addition, namely TT2, TT3 and TT4, or Ge addition (ZF4, ZF5, ZF6 [37]) or Sn addition (ZX4, ZX6, ZX8 [22]) shows that at 800 °C (i.e., in pest regime) Ge and B, respectively, was most and least effective and that at 1200 °C all three elements were not effective as scale spallation was not suppressed. However, only when B was in synergy with Al, Cr and Mo was the oxidation improved compared with the basis alloy JG3 [35], as the alloy TT8 did not pest and its scale did not spall off at 800 and 1200 °C. Thus, the aforementioned synergy of elements in TT8 had the same effect at both temperatures as that of Al and Cr with Ge plus Sn in the alloy OHS1 [23]. In other words, the alloy TT8 had outstanding (for RMICs, and RHEAs and RCCAs) oxidation behaviour at 800 and 1200 °C with density 6.4 g/cm^3^ and specific strength of 377 and 337 MPacm^3^g^−1^, respectively, in AC and HT conditions. Another characteristic difference of the alloys with B or Ge addition [37] with the Sn containing alloys ZX4, ZX6 and ZX8 [22] was the fact that in the latter, tin oxide was rarely (if at all) observed in the scale, in contrast with the former alloys where B_2_O_3_ or GeO_2_ were formed. The latter two oxides form glass with silica.

## 5. Conclusions

We studied the effect of B addition on the density, macrosegregation, microstructure, hardness and oxidation of four RM(Nb)IC alloys, namely the alloys TT2, TT3, TT4 and TT8. In actual fact, these alloys were based on four KZ series alloys (the basis/reference alloys) to which B was added. This choice of alloys made it possible to also compare the effect of B addition on density, hardness or oxidation with that of Ge or Sn addition. 

The addition of B resulted in B macrosegregation that was highest in TT8 and in increased macrosegregation of Si and Ti, both of which were lowest in TT8. With the B addition, the density of the alloys was decreased significantly and was lower than the alloys with Ge addition. The alloy TT8 had the lowest density of 6.41 g/cm^3^ and the highest specific strength at room temperature, which was also higher than that of RCCAs and RHEAs. The Nb_ss_ and T2 silicide were stable in the alloys TT2 and TT3, whereas in TT4 and TT8 the stable phases were the Nb_ss_ and the T2 and D8_8_ silicides. The latter silicide was Al free. The T2 had Si + B + Al ≈ 37.5 at.%, Si/B ≈ 4 and <Nb>/<Si> ≈ 1.7 compared with Si + B ≈ 41.5 at.%, Si/B ≈ 0.5 and <Nb>/<Si> ≈ 1.4 of the D8_8_. Compared with the Ge or Sn addition in the same reference alloy, the B and Ge addition was the least and most effective at 800 °C (i.e., in the pest regime), when no other RM was present in the alloy. Like Ge or Sn, the B addition in TT2, TT3 and TT4 did not suppress scale spallation at 1200 °C. Only the alloy TT8 did not pest and its scales did not spall off at 800 and 1200 °C.

The macrosegregation of Si and Ti, the chemical composition of Nb_ss_ and T2, the microhardness of Nb_ss_ and the hardness of alloys, and the oxidation of the alloys at 800 and 1200 °C were also viewed from the perspective of the alloy design methodology NICE and relationships with the alloy or phase parameters VEC, δ and Δχ. The trends of these parameters and the location of alloys and phases in parameter maps were found to be in agreement with NICE, the one exception being the macrosegregation of Si in TT8 when compared with its basis/reference alloy.

## Figures and Tables

**Table 1:** Nominal and actual composition (at.%) of as cast (AC) and heat-treated (HT) alloys.

Alloy	Condition	Element						
		Nb	Ti	Si	Al	Cr	B	Mo
TT2	nominal	48	24	16	-	5	7	-
-	AC	47.8	24.2	16.3	-	5.2	6.5	-
-	HT	47.3	24	16.5	-	5.3	6.9	-
TT3	nominal	48	24	16	5	-	7	-
-	AC	49.3	23.6	15.4	4.4	-	7.3	-
-	HT	49.5	23.2	15.6	4.4	-	7.3	-
TT4	nominal	40	24	18	5	5	8	-
-	AC	42.4	24.6	15.7	5	5.4	6.9	-
-	HT	40.7	24	17.3	4.8	4.9	8.3	-
TT8	nominal	42.5	24	17	3.5	5	6	2
-	AC	43.4	23	17.2	3.5	4.9	6	2
-	HT	42.9	23.7	17.2	3.3	4.9	5.9	2.1

**Table 2:** Density of alloys, vol.% Nb_ss_, hardness * of alloys, microhardness * of Nb_ss_ and T2 silicide.

Alloy	Hardness HV_alloy_		ρ (g/cm^3^)		Vol.% Nb_ss_		Microhardness HV_phase_			
							Nb_ss_		T2	
	AC	HT	AC	HT	AC	HT	AC	HT	AC	HT
TT2	688 ± 42 622–734	680 ± 28 624–708	6.71	6.72	37	38	472 ± 18 443–496	419 ± 39 377–495	1346 ± 70 1202–1445	1167 ± 84 1015–1265
TT3	630 ± 86 494–757	571 ± 54 501–659	6.52	6.56	38	42	521 ± 34 472–577	379 ± 19 352–411	1232 ± 72 1116–1334	1163 ± 80 1061–1332
TT4	720 ± 70 642–879	607 ± 21 590–659	6.41	6.41	30	39	563 ± 37 503–631	467 ± 15 450–496	1247 ± 55 1172–1334	1239 ± 40 1200–1332
TT8	744 ± 60 673–842	693 ± 28 622–717	6.46	6.44	39	38	531 ± 26 487–576	461 ± 25 417–495	1316 ± 37 1265–1369	1209 ± 48 1143–1299

* given as the average value, standard deviation, and maximum and minimum measured values.

**Table 3:** Macrosegregation (at.%) of solutes MACX (X = Al, B, Cr, Si, Ti) in the cast alloys, where MACX = C_max_^X^ − C_min_^X^ [19].

Alloy	MACX (at.%)				
	Al	B	Cr	Si	Ti
TT2	-	4.2	2.8	3.1	3.2
TT3	2.7	4.2	-	4.5	3.4
TT4	1.1	2.9	1.4	4.7	3.3
TT8	1.1	4.9	1.3	1.3	2.8
TT1 [12]	-	4.4	-	4.1	5.5

**Table 4:** Phases in the as cast (AC) and heat-treated (HT) alloys according to the XRD and EPMA data.

Alloy and Condition	Phase								
	Nb_ss_	T2	Cr & Ti Rich Phase	Ti Rich Phase	C14-Laves	Nb_3_Si	D8_8_	Nb_ss_ + T2 Eutectic	TiN
TT2-AC	X *	X *	X	X	X	X	-	X	-
TT2-HT ^◊^	X	X *	-	-	-	-	-	-	X °
TT3-AC	X *	X *	-	-	-	-	-	X	-
TT3-HT ^+^	X	X *	-	-	-	-	-	-	-
TT4-AC	X *	X *	-	-	-	-	X	X	-
TT4-HT ^+^	X	X	-	-	-	-	X	-	-
TT8-AC	X *	X *	-	-	-	X	X	X	-
TT8-HT ^+^	X	X *	-	-	-	-	X	-	X °

* indicates the presence also of Ti-rich phase (see Table 5), ^+^ 1500 °C/100 h, ^◊^ 1200 °C/100 h, ^o^ indicates that alloy was contaminated by nitrogen.

**Table 5:** EPMA data for the chemical composition (at.%) of phases in AC and HT alloys.

Alloy and Condition	Phase	Element						
		Nb	Ti	Si	Al	B	Cr	Mo
TT2-AC	Nb_ss_	57.2–63.2 60.3 ± 2.4	23.7–29 26.6 ± 1.7	1.4–1.9 1.6 ± 0.2	-	3.6–6.3 5 ± 0.2	5.3–7.9 6.5 ± 1	-
-	Ti rich Nb_ss_	38.8–46.6 42.5 ± 3.8	36.9–43.9 40.7 ± 1.7	1.6–3.5 2.4 ± 0.2	-	0.6–3.3 1.8 ± 0.2	10.1–14.1 12.6 ± 2.3	-
-	Phase rich in Cr & Ti	14.8–21.9 18.9 ± 2.6	40.2–53.9 48.3 ± 1.4	3.4–12.9 7.1 ± 2.7	-	-	23.4–28.5 25.7 ± 4.5	-
-	T2	46–48.1 47.1 ± 0.6	13.4–15.4 14.3 ± 0.9	23.4–26.4 25.3 ± 1	-	10.9–14.9 12.9 ± 1.1	0.2–0.7 0.4 ± 0.1	-
-	Ti rich T2	39.1–43.7 41.6 ± 1.2	18.6–23.8 20.8 ± 1.6	28.1–30.3 29.3 ± 0.7	-	5.9–9.4 7.8 ± 1	0.2–0.8 0.5 ± 0.1	-
TT3-AC	Nb_ss_	52.9–59.4 55.8 ± 0.6	28.6–32.6 30.6 ± 1.2	1.6–2 1.8 ± 0.1	3.9–8.4 6.8 ± 2.2	4–6 5 ± 0.6	-	-
-	Ti rich Nb_ss_	43.4–49.9 47.7 ± 0.6	38.7–47.8 42.7 ± 1.6	1.7–2 1.9 ± 0.1	3.3–5.4 4.5 ± 2.4	1.9–4.4 3.2 ± 0.7	-	-
-	T2	46–48.3 46.9 ± 0.4	13.3–15.6 14.4 ± 0.2	22.5–26.7 25 ± 1.2	0.4–1.2 0.8 ± 0.1	10.8–16.2 12.9 ± 1.5	-	-
-	Ti rich T2	41.3–45.1 42 ± 1.4	18.2–19.9 19 ± 0.3	29.1–30.4 29.8 ± 0.4	0.6–2.7 1.7 ± 0.6	6.6–8.6 7.5 ± 0.5	-	-
TT4	Nb_ss_	46.4–50.1 48.3 ± 2.1	29.4–31.6 30.6 ± 1.7	1.2–1.7 1.4 ± 0.1	7.1–7.7 7.4 ± 0.3	2.5–3.9 3.3 ± 0.3	7.9–10.1 9 ± 0.4	-
-	Ti rich Nb_ss_	32.7–39.4 35.6 ± 1.1	37–42.5 39.7 ± 0.5	1.1–1.5 1.3 ± 0.1	6.7–7.3 7 ± 0.4	0.3–1.2 0.7 ± 0.4	14.1–16.9 15.7 ± 1.4	-
-	T2	40.5–42.6 41.4 ± 0.3	19–20.3 19.7 ± 0.4	27.5–29.9 29.1 ± 0.8	1.7–2.7 2.3 ± 0.3	5.2–8.3 6.8 ± 0.4	0.4–1.1 0.7 ± 0.1	-
-	Ti rich T2	28.5–35.8 32.5 ± 1	26.2–33.5 29.3 ± 0.9	27.4–29.3 28.3 ± 0.8	3.7–4.3 4 ± 0.3	3.2–5.5 4.4 ± 0.4	0.8–2.2 1.5 ± 0.4	-
-	D8_8_	44.5–46.9 45.9 ± 0.6	11.7–13.3 12.5 ± 0.6	14.4–16 15 ± 0.8	-	23.5–27 25.6 ± 0.4	0.6–1.4 1 ± 0.3	-
TT8-AC	Nb_ss_	42.4–49.9 45.9 ± 2.5	26.9–30.9 28.8 ± 1.1	1.1–1.5 1.3 ± 0.3	6.8–8.5 7.6 ± 0.4	0–5.4 1.8 ± 1.4	9.1–11.5 10.6 ± 1.4	3.5–4.5 4 ± 0.6
-	Ti rich Nb_ss_	30.8–38 34.5 ± 0.7	36.4–41.5 38.4 ± 2.1	1–1.4 1.2 ± 0.3	6–7.3 6.8 ± 0.7	0–3.5 1.1 ± 0.8	14.2–16.6 15.6 ± 2.2	2–2.8 2.4 ± 0.1
-	T2	36.7–43.5 40.5 ± 1.2	18.2–20.8 19.1 ± 0.4	24–28 25.6 ± 4.9	2.6–3.5 2.9 ± 0.2	6.7–15.5 10.4 ± 6.3	0.6–1.1 0.8 ± 0.2	0.6–0.7 0.7 ± 0.1
-	Ti rich T2	29.2–32.1 30.7 ± 1.3	29.4–31.5 30.2 ± 0.9	24.7–27.8 26.4 ± 3.5	3–4.2 3.3±0.5	6.5–9.5 8 ± 1.1	0.3–1 0.9 ± 0.3	0.3–0.7 0.5 ± 0.2
-	D8_8_	41.9–45.2 44.7 ± 2	9.2–12.3 10.2 ± 0.6	11–13.5 12.4 ± 0.9	-	26.4–33.7 30 ± 2.6	0.8–1.3 1 ± 0.2	1.6–1.8 1.7 ± 0.2
TT2-HT	Nb_ss_	52.1–55.2 53.2 ± 1.2	30.1–33.6 31.9 ± 1.6	0.3–0.5 0.4 ± 0.1	-	2–4.3 3.2 ± 0.5	10.2–12 11.3 ± 0.2	-
-	T2 *	46.9–48.6 47.8 ± 1.2	13.7–15.2 14.4 ± 1	23.3–26 24.5 ± 1.2	-	10.7–14.8 12.7 ± 1.5	0.3–0.8 0.6 ± 0.1	-
TT3-HT	Nb_ss_	54.8–57.9 56.4 ± 1	29.2–32 30.7 ± 1	0.3–0.5 0.4 ± 0.1	7.5–8.3 7.8 ± 0.6	3.8–5.6 4.7 ± 0.6	-	-
-	T2	41.8–46.2 44.1 ± 2.4	16.1–19.7 17.4 ± 2.2	25.4–28 26.9 ± 2	0.8–1.8 1.4 ± 0.9	9–11.7 10.2 ± 2.6	-	-
-	Ti rich T2	34–36.6 34.8 ± 1.3	25–29 27.4 ± 1	28–29.5 28.9 ± 0.7	3.7–4.5 4.2 ± 0.5	3.8–5.9 4.7 ± 0.4	-	-
TT4-HT	Nb_ss_	48.5–50.5 49.8 ± 0.8	28.1–29.5 28.6 ± 0.6	0.4–0.7 0.5 ± 0.1	7.3–7.8 7.6 ± 0.2	1.5–4.3 2.8 ± 1	10.4–11 10.7 ± 0.2	-
-	T2	35.8–40 37.7 ± 1.9	19.7–24.6 22.3 ± 1.9	29.4–31.2 30.2 ± 0.8	2.3–3.8 3.3 ± 0.5	4.2–6.3 5.5± 0.8	0.7–1.4 1 ± 0.2	-
-	D8_8_	42.7–44.5 43.6 ± 0.8	11.6–12.4 11.9 ± 0.3	12.9–14.2 13.4 ± 0.5	-	28–30.7 29.2 ± 1	1.6–2.2 1.9 ± 0.3	-
TT8-HT	Nb_ss_	46.2–48.7 45.6 ± 1	23.9–31.5 29.8 ± 2.2	0.4–0.6 0.5 ± 0.1	7.2–8.2 7.6 ± 0.5	0–4.8 1 ± 0.8	10.5–11.8 11.2 ± 0.7	4.1–4.4 4.3 ± 0.1
-	T2	35.8–44.5 41 ± 4.6	18.7–21.5 20.1 ± 1.4	27.1–33.2 29.3 ± 3.4	2.7–4.3 3.3 ± 0.8	0–8.7 4.7 ± 4.4	0.4–1.9 1.1 ± 0.8	0.3–0.7 0.5 ± 0.2
-	Ti rich T2	30.9–33.7 31.6 ± 1.2	25.9–31.2 28.1 ± 2	26.4–29.6 28 ± 1.3	3.3–3.6 3.5 ± 0.2	3.3–11 6.8 ± 4.6	1.1–1.8 1.5 ± 0.3	0.4–0.5 0.5 ± 0.02
-	D8_8_	41.2–48.9 44.2 ± 4.3	11.4–12.6 12.1 ± 0.7	12.4–4.9 13.5 ± 1.5	-	19.2–31 26.5 ± 6.4	1.5–2.3 2 ± 0.5	1.6–1.9 1.7 ± 0.1

* see text.

**Table 6:** Oxidation rate constants and mass changes of the alloys after isothermal oxidation for 100 h at 800 and 1200 °C.

Alloy	Oxidation Rate Constant		Mass Change ΔW/A (mg/cm^2^)		Pest Oxidation	Scale Spallation	
	800 °C k_l_ (g/cm^2^s)	1200 °C k_p_ (g^2^/cm^4^s)	800 °C	1200 °C	800 °C	800 °C	1200 °C
MASC	1.53 × 10^−7^	1.58 × 10^−8^	36 (≤65 h)	80	MC *	-	Yes
TT1	1.86 × 10^−7^	1.53 × 10^−8^	63	80.3	No	Yes	Yes
TT2	1.89 × 10^−7^	3.32 × 10^−8^	61.4	105.6	No	Yes	Yes
TT3	1.22 × 10^−7^	1.21 × 10^−8^	38.6	66.1	No	Yes	Yes
TT4	8.9 × 10^−8^	3.4 × 10^−9^	27.9	34	No	Yes	Yes
TT8	3 × 10^−9^	3.3 × 10^−9^	1.4	30	No	No	No

* MC = Maltese cross.

**Table 7:** Comparison of the macrosegregation (MACX) of X = B, Si or Ti in the B containing alloys of this work and their basis/reference alloys KZ3, KZ4, KZ5, KZ7 [16] and JG3 [17] (see Appendix A). Large button ingots (0.6 kg) of all alloys were prepared using arc melting. Note that the table includes data for the alloy TT1 (Nb-24Ti-18Si-8B [12]) and its basis alloy KZ3 (Nb-24Ti-18Si [16]).

Alloy	MACSi (at.%)		Alloy	MACB (at.%)		Alloy	MACTi (at.%)	
TT4	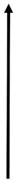	4.7	TT8	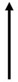	4.9	TT1	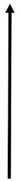	5.5
TT3		4.5	TT1		4.4	TT3		3.4
TT1		4.1	TT2		4.2	TT4		3.3
TT2		3.1	TT3		4.2	TT2		3.2
JG3		2.6	TT4		2.9	KZ3		3.1
KZ7		2.3	-		-	TT8		2.8
KZ4		1.9	-		-	KZ7		2.3
KZ3		1.6	-		-	JG3		1.5
TT8		1.3	-		-	KZ4		1.4
KZ5		1.3	-		-	KZ5		1.4

## Data Availability

All the data for this paper is given in the paper and its Supplementary Materials, other data cannot be made available to the public.

## Supplementary Materials

The following are available online at https://www.mdpi.com/article/10.3390/ma14206101/s1, Figure S1: X-ray diffractograms (a), (c), (e) and (g) of AC alloys TT2, TT3, TT4 and TT8, respectively, and (b), (d), (e) and (h) of HT alloys TT2, TT3, TT4 and TT8, respectively; Figure S2: X-ray diffractograms of oxide scale formed on the alloy TT2 (a) at 800 °C and (b) at 1200 °C.

## Appendix A

(A1) $$\delta =100\sqrt{\overset{n}{\underset{n=1}{\sum }}c_{i\;}{\left(1-r_{i}/\bar{r}\right)}^{2}}$$

(A2) $$\Delta \chi =\sqrt{\overset{n}{\underset{n=1}{\sum }}c_{i\;}{\left(\chi_{i}-\;\bar{\chi }\right)}^{2}}$$

(A3) $$VEC=\overset{n}{\underset{n=1}{\sum }}c_{i\;}{\left(\mathrm{VEC}\right)}_{i}$$

(A4) $$\bar{r}=\overset{n}{\underset{n=1}{\sum }}c_{i}\;r_{i}$$

(A5) $$\bar{\chi \;}=\overset{n}{\underset{n=1}{\sum }}c_{i}\;\chi_{i}$$

(A6) $$\Delta H_{mix}=\overset{n}{\underset{i=1,j\neq 1}{\sum }}\Pi_{ij}\;c_{i}\;c_{j}\;$$

(A7) $$\Pi_{ij}=4{\Delta_{mix}}^{AB}$$

(A8) $$\Delta S_{mix}=-R\overset{n}{\underset{i=1}{\sum }}c_{i}\;\ln c_{i}$$

(A9) $$T_{m}=\overset{n}{\underset{i=1}{\sum }}c_{i}\;T_{m_{i}}$$

where *c_i_*, *r_i_*, *χ_i_*, (*VEC*)*_i_* and *T_mi_*, respectively, are atomic percentage, atomic radius, Pauling electronegativity, VEC and melting point of *i*th element, ${\Delta_{mix}}^{AB}\;$is the mixing enthalpy of binary liquid *AB* alloy and *R* is the gas constant.

**Table A1:** Nominal compositions (at.%) of reference alloys used in this work.

Alloy				Element						
	Nb	Ti	Si	Al	Cr	Mo	B	Ge	Sn	Ref.
JG3	bal.	24	18	5	5	2	-	-	-	[17]
KZ3	bal.	24	18	-	-	-	-	-	-	[16]
KZ4	bal.	24	18	-	5	-	-	-	-	[16]
KZ5	bal.	24	18	5	5	-	-	-	-	[16]
KZ7	bal.	24	18	5	-	-	-	-	-	[16]
OHS1	bal.	24	18	5	5	-	-	5	5	[23]
TT1	bal.	24	18	-	-	-	8	-	-	[12]
ZF3	bal.	24	18	-	-	-	-	5	-	[33]
ZF4	bal.	24	18	-	5	-	-	5	-	[30]
ZF5	bal.	24	18	5	-	-	-	5	-	[31]
ZF6	bal.	24	18	5	5	-	-	5	-	[32]
ZX4	bal.	24	18	-	5	-	-	-	5	[22]
ZX6	bal.	24	18	5	-	-	-	-	5	[22]
ZX8	bal.	24	18	5	5	-	-	-	5	[22]

**Table A2:** <Nb>/<Si> and Si/B ratios and Si + B and Si + B + Al sums in T2 and D88 silicides in AC and HT alloys, where <Nb> = Nb + TM + RM, <Si> = Al + B + Si, TM = Cr, Ti and RM = Mo.

Alloy and Condition	Phase	Parameter			
		Si + B (at.%)	Si/B	Si + B + Al (at.%)	<Nb/<Si>
TT2-AC	T2	38.2	2	-	1.6
-	Ti rich T2	37.1	3.8	-	1.7
TT2-HT	T2	37.2	1.9	-	1.7
-	Ti rich T2 *	34.1 to 37 Average 35.6	1, 3.8, 6.3 Average 3.7	-	1.7 to 1.9 Average 1.8
TT3-AC	T2	-	1.9	38.6	1.6
-	Ti rich T2	-	4	39	1.56
-	T2 very rich in Ti	-	5	37.5	1.67
TT3-HT	T2	-	2.6	38.5	1.6
-	Ti rich T2	-	6.1	37.7	1.65
TT4-AC	T2	-	4.3	38.2	1.62
-	Ti rich T2	-	6.4	36.7	1.72
-	D8_8_	40.8	0.6	-	1.45
TT4-HT	T2	-	5.5	39	1.56
-	D8_8_	42.7	0.5	-	1.3
TT8-AC	T2	-	2.5	38.9	1.57
-	Ti rich T2	-	3.3	37.7	1.65
-	D8_8_	42.6	0.4	-	1.35
TT8-HT	T2	-	6.2	37.3	1.68
-	Ti rich T2	-	4.1	38.3	1.61
-	D8_8_	40	0.5	-	1.5

* see text.
