# Supplementary material for: The Effect of Boron on the Microstructure and Properties of Refractory Metal Intermetallic Composites (RM(Nb)ICs) Based on Nb-24Ti-xSi (x = 16, 17 or 18 at.%) with Additions of Al, Cr or Mo
Source: Materials (Basel). 2021 Oct 15;14(20):6101. doi: 10.3390/ma14206101 (PMC8537113; doi:10.3390/ma14206101)
Supplement: Supplementary file 1 [file materials-14-06101-s001.zip › materials-1369782-supplementary.pdf]

materials

Supplementary Materials

The Effect of Boron on the Microstructure and Properties of Refractory Metal Intermetallic Composites (RM(Nb)ICs) Based on Nb-24Ti-xSi (x = 16, 17 or 18 at.%) with Additions of Al, Cr or Mo

Tophan Thandorn [1] and Panos Tsakiropoulos [2,*]

[1] Department of Materials Science and Engineering, School of Science, Mae Fah Luang University, Chiang Rai 57100, Thailand; tophan@mfu.ac.th

[2] Department of Materials Science and Engineering, Sir Robert Hadfield Building, The University of Sheffield, Sheffield S1 3JD, UK

* Correspondence: p.tsakiropoulos@sheffield.ac.uk

Supplementary Materials

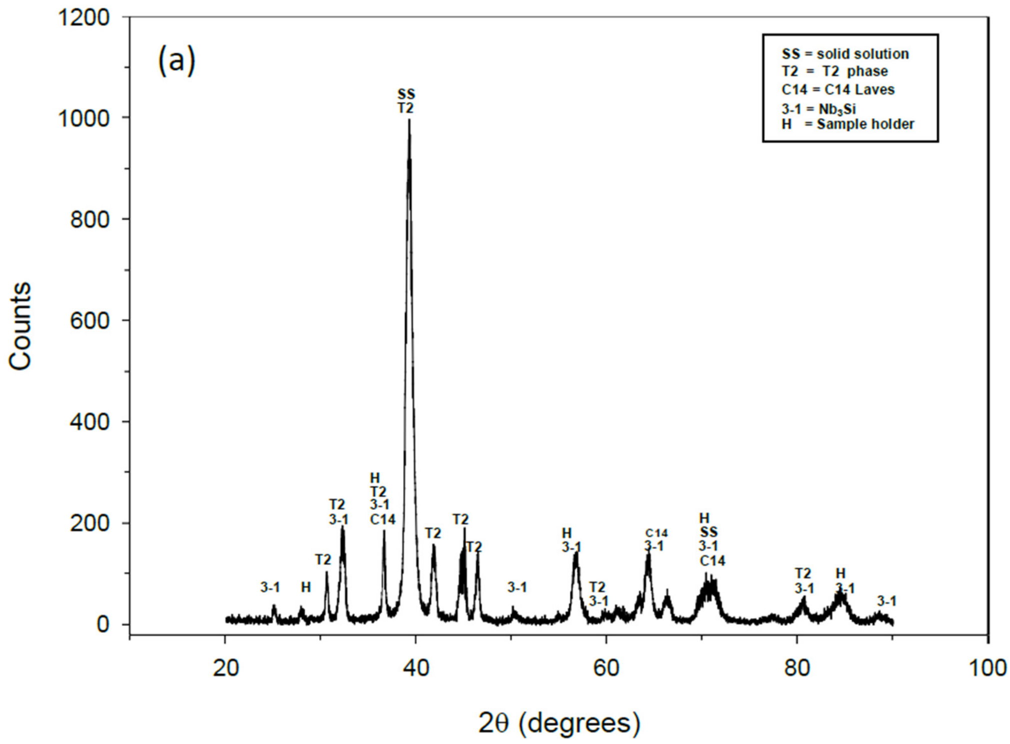

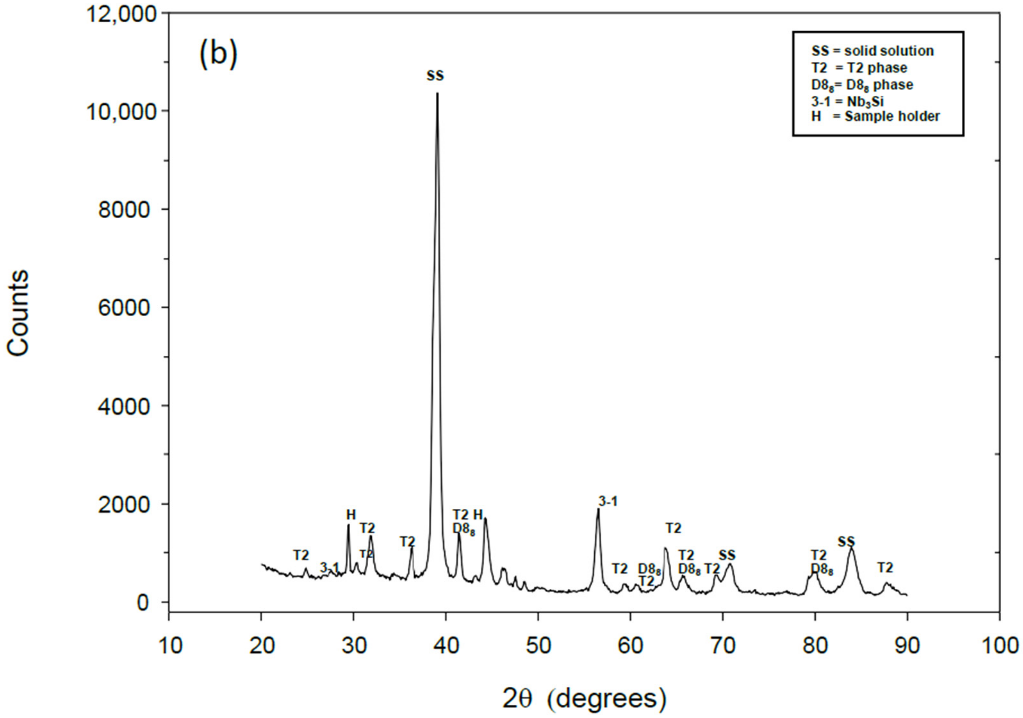
(b)
SS = solid solution
T2 = T2 phase
D8$_8$= D8$_8$ phase
3-1 = Nb_3Si
H = Sample holder
Counts
2θ (degrees)

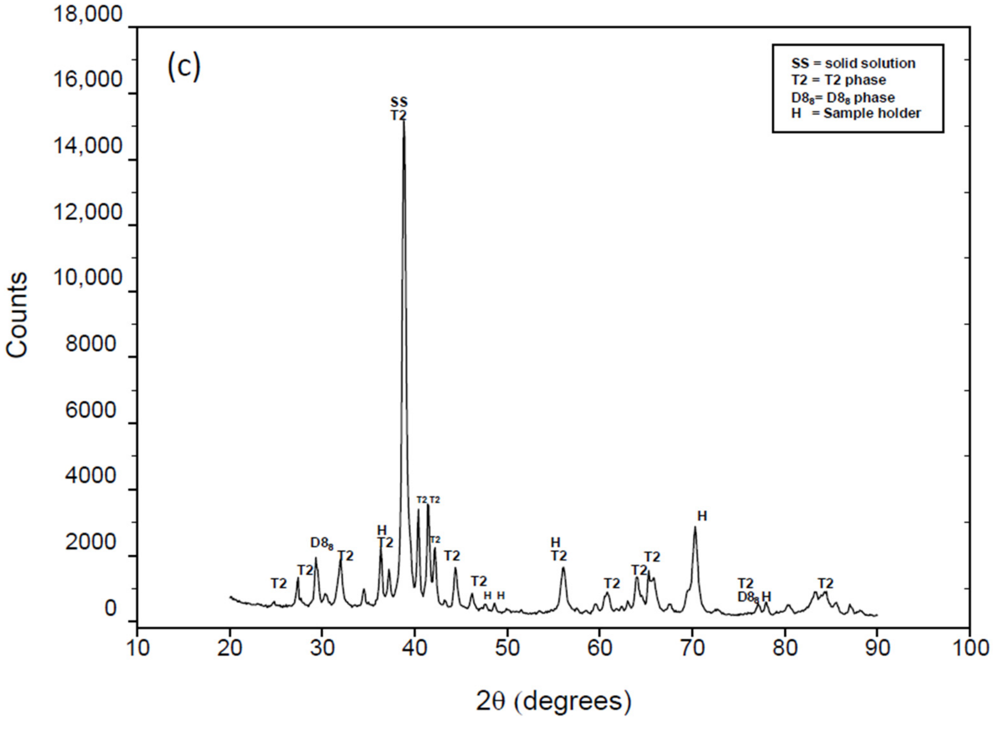
(c)
SS = solid solution
T2 = T2 phase
D8$_8$= D8$_8$ phase
H = Sample holder
Counts
2θ (degrees)

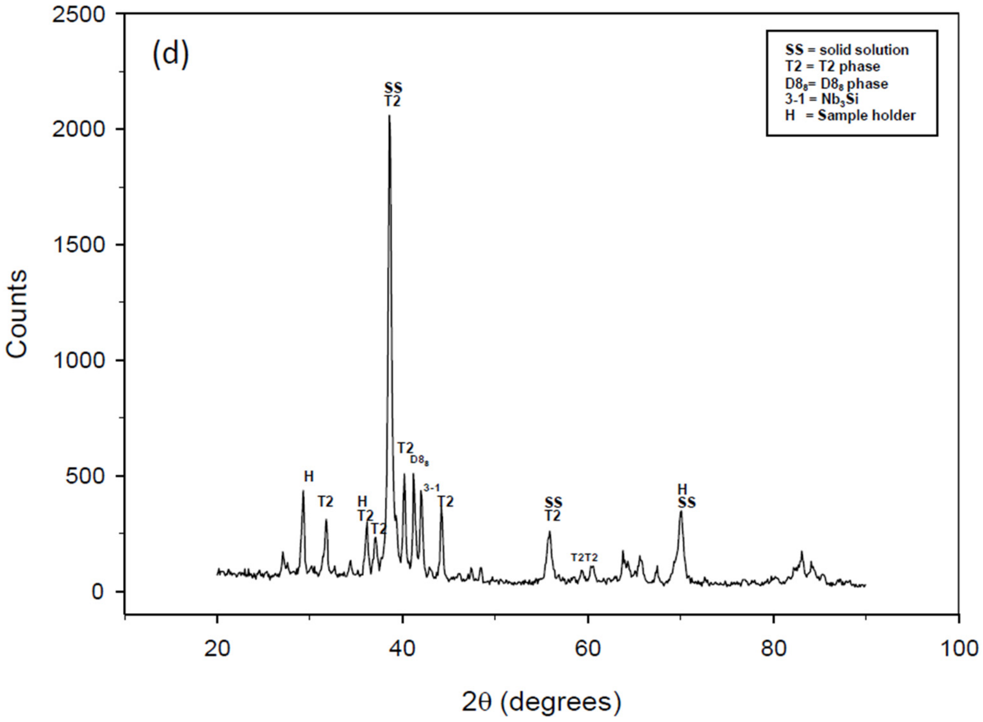
(d)
SS = solid solution
T2 = T2 phase
D8₈= D8₈ phase
3-1 = Nb₃Si
H = Sample holder
Counts
2θ (degrees)
SS T2
H
T2
D8₈
3-1
SS
T2T2

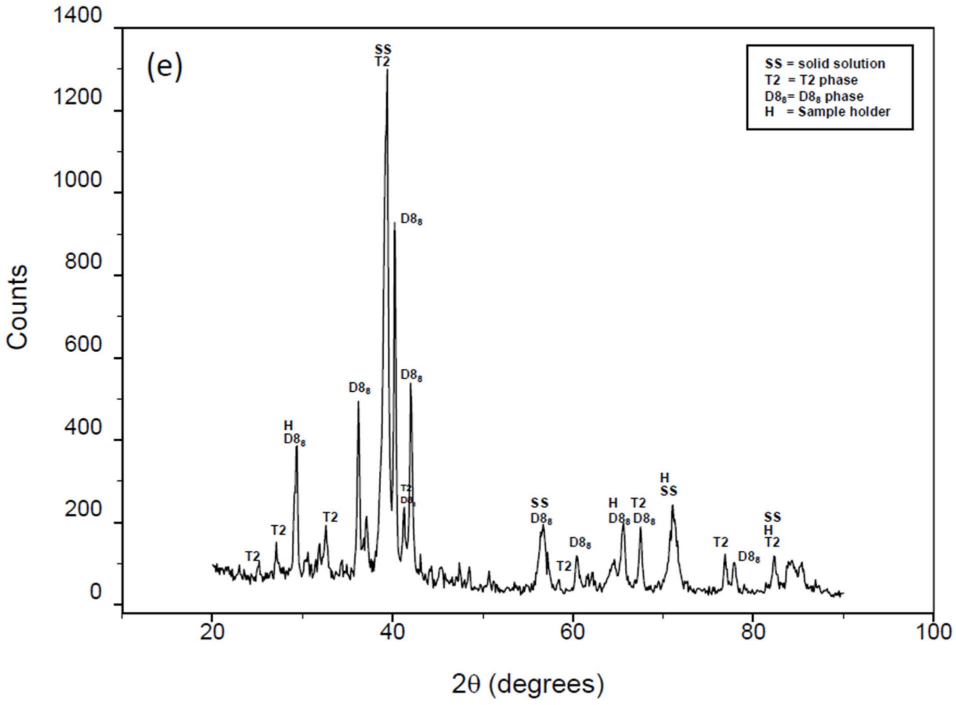
(e)
SS = solid solution
T2 = T2 phase
D8₈= D8₈ phase
H = Sample holder
Counts
2θ (degrees)
SS T2
D8₈
H D8₈
SS
H SS

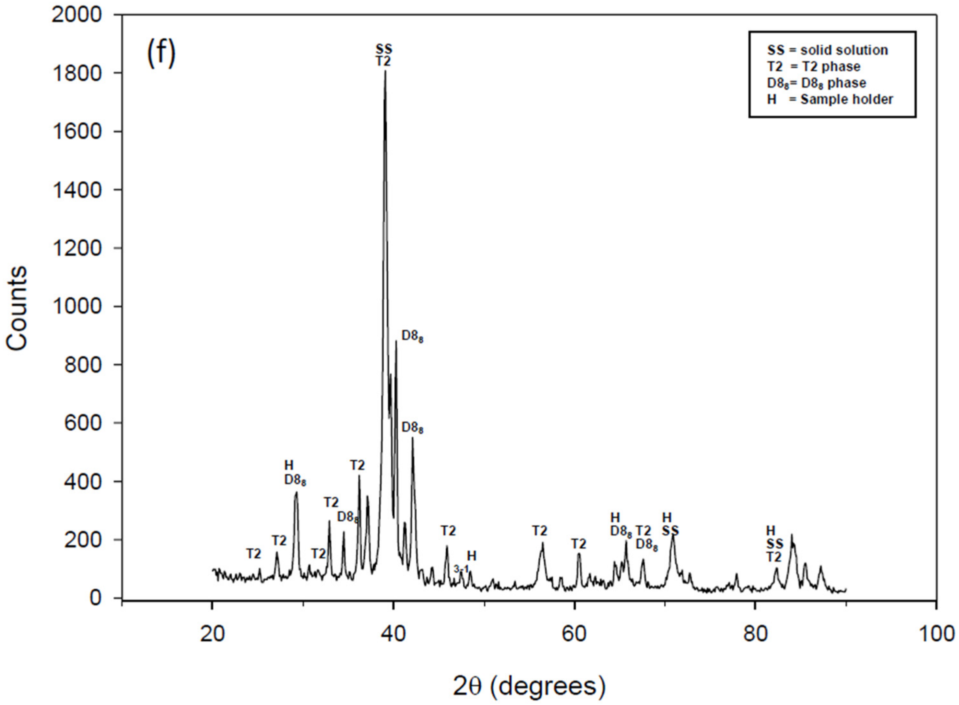
(f)
SS = solid solution
T2 = T2 phase
D8₈= D8₈ phase
H = Sample holder
Counts
2θ (degrees)

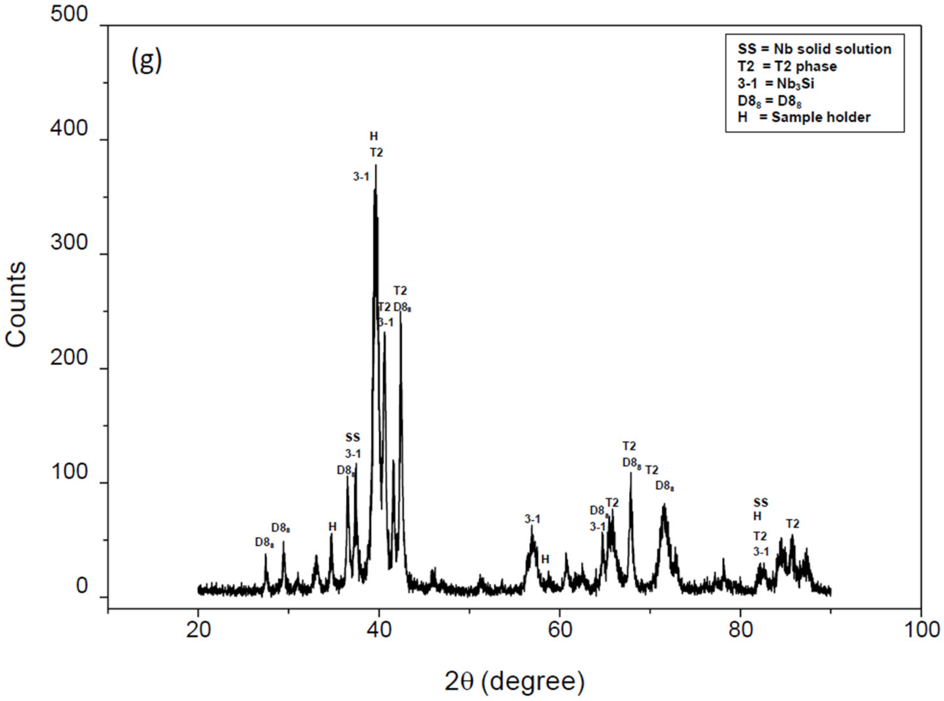
(g)
SS = Nb solid solution
T2 = T2 phase
3-1 = Nb₃Si
D8₈ = D8₈
H = Sample holder
Counts
2θ (degree)

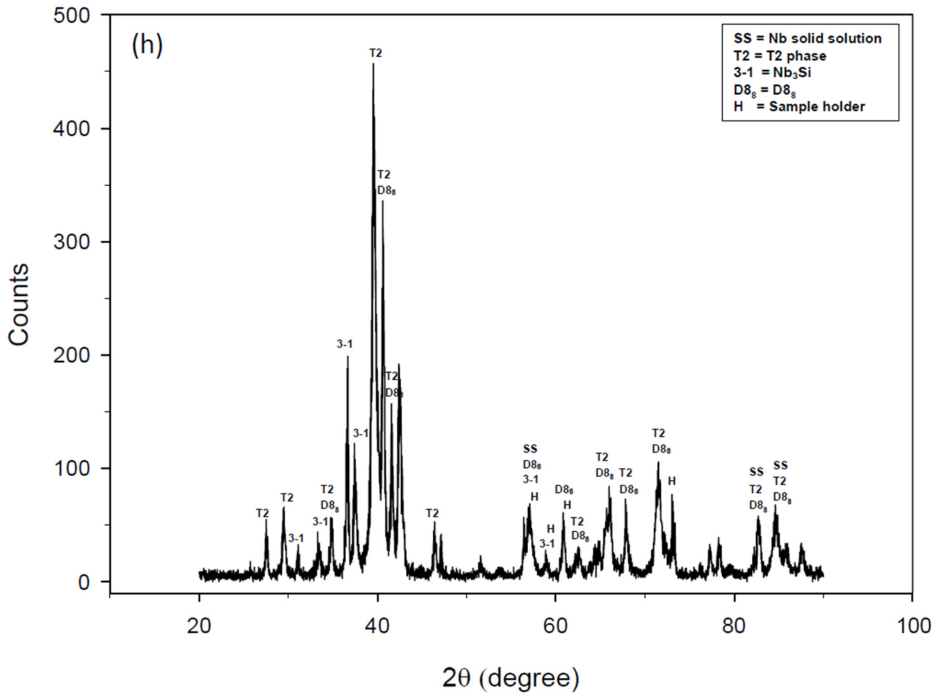

Figure S1. X-ray diffractograms (**a**), (**c**), (**e**) and (**g**) of AC alloys TT2, TT3, TT4 and TT8, respectively, and (**b**), (**d**), (**e**) and (**h**) of HT alloys TT2, TT3, TT4 and TT8, respectively.

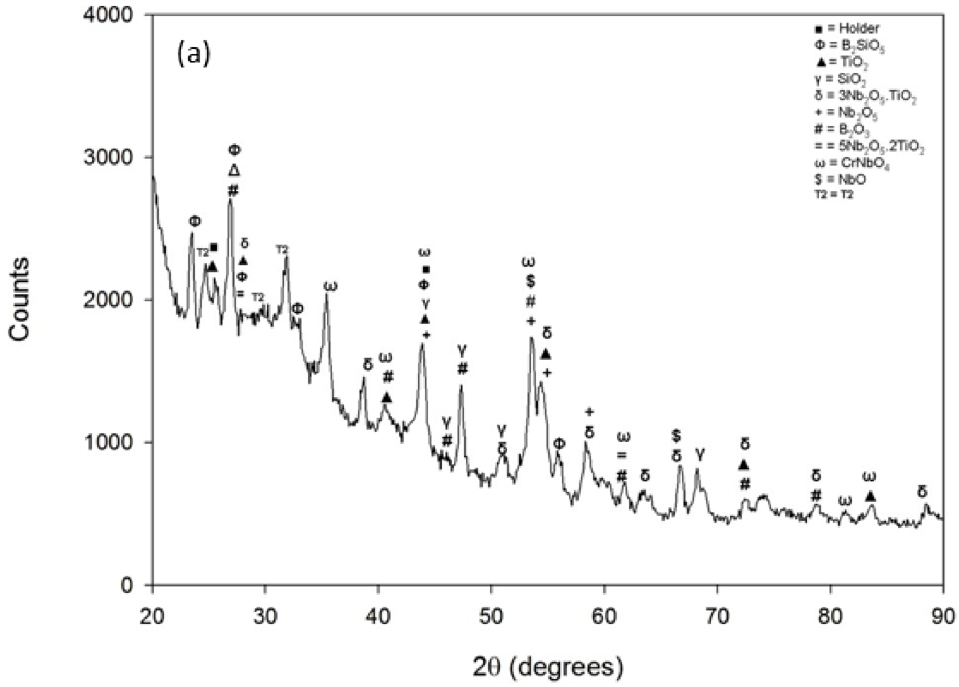

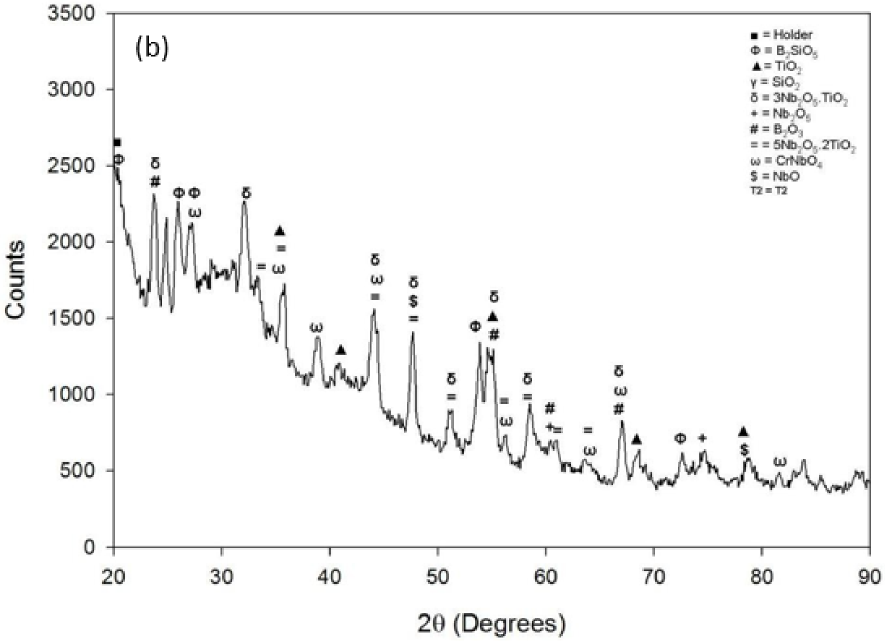

Figure S2. X-ray diffractograms of oxide scale formed on the alloy TT2 (**a**) at 800 °C and (**b**) at 1200 °C.
